# YOLO-SOD: Color probability map-based fuzzy framework using deep models for face detection

**DOI:** 10.1371/journal.pone.0360794

**Published:** 2026-10-22

**Authors:** Aniqa Saba, Muhammad Faizan Shirazi, Yawar Rehman, Muhammad Mubashir Khan

**Affiliations:** 1 Department of Electronic Engineering, Faculty of Electrical and Computer Engineering. NED University of Engineering & Technology, Karachi, Pakistan; 2 Department of Computer Science & Information Technology, Faculty of Electrical and Computer Engineering. NED University of Engineering & Technology, Karachi, Pakistan; 3 Institute for Advanced Technology Convergence (Mobility), Kyungpook National University, Buk-gu, Daegu, Republic of Korea; National University of Sciences and Technology, PAKISTAN

## Abstract

Detecting small faces remains challenging due to scale variation and limited discriminative cues at low resolutions. We address this with a two-pronged approach. First, we modify YOLOv5 by adding extra convolutional layers and small-object–focused detection heads that operate on larger, higher-resolution backbone feature maps, improving sensitivity to tiny targets. Second, we introduce a dynamic, image adaptive patching strategy for scale selection. We compute Cr channel color probability maps and measure the number and density of pixel clusters; a Fuzzy Inference System (FIS) uses these cues to select the most suitable patch size for near and far faces. On the WIDER FACE benchmark, our method attains 93.07% mAP@50 (Easy), 91.25% mAP@50 (Medium), and 84.45% mAP@50 (Hard). On our proposed local dataset, the proposed model achieves 49.36% mAP@50 for different size faces. Beyond faces, the approach generalizes to small object detection on VisDrone-DET2019, reaching 46.76 mAP@50. These results demonstrate that coupling small-object-aware architecture design with color-guided, fuzzy patch selection yields competitive mAP@50 for small and distant targets.

## Introduction

Deep networks for computer vision have emerged as a dynamically evolving domain of critical importance, enabling machines to interpret and process visual information from images and video streams [1]. One key area of focus within this realm is face detection, which has gained widespread attention due to its critical applications in identity verification, surveillance, access control, and user interaction.

Given the ubiquity of facial imagery in digital content, researchers have curated extensive public datasets to facilitate the training and evaluation of deep learning models [2,3]. Consequently, modern deep learning-based face detectors have achieved remarkable performance, with many surpassing 80% mAP@50 on benchmark datasets, especially under controlled conditions [4–6].

In publicly available datasets, people are usually shown posing for images, and their faces are typically directed toward the camera lens. These are considered easy cases, and the mean average precision of deep learning detectors easily exceeds 80%. It can be inferred that these are controlled settings without pose changes, directional variations, or occlusions. Therefore, we argue that the current state-of-the-art deep models that give 80% mAP@50 may exhibit degraded performance to detect faces in the wild, i.e., without controlled settings [2,3].

In our paper, we propose a dataset that was recorded in the wild, without any preconditions for accepting the images, so that original real-world conditions can be absorbed as much as possible. The dataset was uniformly sampled from recorded videos. Our proposed dataset includes persons with near, medium, and far faces, making it suitable for detector evaluation under real conditions.

Despite the remarkable advancements in face detection, largely propelled by the rapid evolution of convolutional neural networks, challenges persist, particularly in achieving a balance between speed and accuracy, especially when dealing with small-scale and densely packed faces [7]. Moreover, real-world object detection methods often grapple with limitations in accuracy, speed, and overall efficiency [1]. The You Only Look Once series of detectors has emerged as a prominent architecture in the field of object detection, prized for its real-time processing capabilities and suitability for a wide array of applications [8]. These models, including the recent YOLOv8, have demonstrated effectiveness in various domains, including traffic management and autonomous vehicles, though detecting diminutive objects in cluttered scenes from afar continues to pose a significant hurdle [9].

However, YOLO-style detectors predict bounding boxes from grid-based feature maps at multiple scales. When many small objects occupy a limited image region, their centers may fall within the same or neighboring grid cells, making assignment and localization more difficult. One solution is to increase the number of grid cells, but the memory and computation required for small objects make this style of object detection slow. Creating image patches is one possible solution; however, the effectiveness of fixed-size patches deteriorates when small, medium, and large objects occur in the same dataset, as in face detection.

Therefore, to solve this problem, we propose image-adaptive patches using Cr-channel color probability maps. This allows us to consider a different number of patches for every image, depending on the information present in the Cr channel.

Challenges also arise due to the intricacies involved in capturing complex features of small targets, especially in environments like those encountered with drone imagery [10]. Recent research endeavors have focused on enhancing YOLO models to address these challenges, adapting techniques such as dilated convolutions and attention mechanisms to improve feature extraction and detection accuracy [11].

To address this challenge, we modified the YOLOv5 architecture by adding dedicated convolutional layers and detection heads that are specifically responsible for small object detection. These layers are attached at the earlier stages of the network to reduce the loss of small-object information caused by downsampling inside the deeper layers.

Our study suggests that face detection evaluation should consider model resilience under different environments and settings, such as distance from the camera, face angle from the camera’s perspective, and the effect of headgear, caps, masks, etc. on detection accuracy. Secondly, there is a need for a dedicated deep network framework for small object detection. Thirdly, the patching technique should be modified so that the image can be divided based on small-object density for each image.

To address this problem, we present the following contributions.

1. We modified the YOLOv5 architecture by adding extra convolutional layers and detection heads dedicated to small-object detection. The feature maps are taken from the earlier layers inside the backbone and the additional head is directly connected to those features for learning. This helps the deep model to understand small object features before they are diminished by downsampling in the deeper network layers.
2. We introduced an innovative approach to adapting patch sizes dynamically for face detection from different distances using Cr channel color probability maps. These maps supply important information cues on the number and density of clusters of pixels, which can be used to estimate the suitable patch size. Then based on the information of patch size,’N’ number of patches can be generated inside the input image for the detection of faces at different distances. A Fuzzy Inference System (FIS) was also designed to find the best number of patches for each image for face detection.
3. We proposed a dataset (recorded locally) for the evaluation of face detection models under real conditions. The proposed dataset was recorded in the wild and in an uncontrolled environment. This dataset helped us to evaluate the detection resilience of the proposed method and state-of-the-art detection methods.

The remainder of the paper is organized as follows. The Related work section reviews the most recent and relevant literature. The Proposed methodology section details the proposed approach. The Results section presents and discusses the experimental findings. Finally, the Conclusion section summarizes the study.

## Related work

Face detection stands as a fundamental application within computer vision. Although research in this domain began years ago, a major breakthrough came with the pioneering work of Viola and Jones. They introduced a method utilizing rectangular Haar-like features, boosted feature selection, and cascaded classifiers, which was the first to demonstrate practical viability for real-world use, including deployment on hardware such as mobile phones. Since their contribution, the field has seen remarkable advancements, with the introduction of more sophisticated algorithms capable of detecting faces in a wide range of scenarios. Nonetheless, detecting faces “in the wild”—that is, in unconstrained environments—remains a significant challenge due to varying factors such as changes in orientation, occlusions, facial accessories, lighting conditions, and weather variations. These complexities continue to make robust face detection a difficult task in real-world settings.

In recent years, numerous deep learning-based techniques and their variants have been introduced to address the challenges associated with face detection in unconstrained environments. Among the earliest advancements in this direction, the year 2016 marked the emergence of high-performing detectors. For instance, the authors in [12] applied Faster R-CNN to the problem of face detection using region-based convolutional strategies. While effective, the model struggled with detecting small and occluded faces. Further efforts from researchers in [13–16] introduced state-of-the-art networks that improved accuracy but were slower in comparison to single-shot detectors and still faced challenges in identifying small-scale faces.

In 2017, Najibi et al. [17] proposed a single-stage headless network aimed at real-world face detection. However, this model also underperformed on small and occluded faces. The detection of small-sized faces within high-resolution images remains a significant hurdle in this domain. Although several powerful detectors have been released online [18–21], they exhibit notably reduced performance in the presence of pose variation, occlusions, and small face regions. In response to these limitations, deeper networks were introduced [22–26], achieving better results on benchmark datasets at the cost of increased computational demands.

To address issues of inference speed and model size, lightweight architectures [27] were proposed. Additionally, transformer-based object detectors and backbones [28–31] demonstrated enhanced detection accuracy, while higher-resolution networks [32] were developed to improve performance under diverse conditions. EfficientDet [33], introduced in 2020, offered a balanced trade-off between accuracy and computational efficiency through compound model scaling and multiscale feature fusion on the Microsoft COCO dataset [34]. Another notable approach, RetinaFace [4], is capable of detecting faces at multiple scales and orientations. By leveraging multi-task supervision for face boxes, landmarks, and dense face information, it achieved 91.4% mAP@50 on the WIDER FACE Hard test subset [2].

CenterFace [35] was designed for real-time image and video processing and is suitable for deployment in resource-constrained environments due to its lightweight design. DSFD (Dual Shot Face Detector) [5] uses dual-shot feature enhancement and reported 96.3%, 95.4%, and 90.1% mAP@50 on the Easy, Medium, and Hard WIDER FACE validation subsets, respectively. FaceBoxes [36] targets CPU real-time face detection and was evaluated on the FDDB benchmark [37]. PyramidBox [38] uses contextual information and feature pyramids to improve difficult face detection.

In 2021, the High-Low Adaptation model HLA-Face [39] demonstrated strong low-light face detection performance, achieving 44.4% mAP@50 on the DARK FACE benchmark. Another notable contribution, RefineFace [40], was proposed in 2021 and incorporates five modules including two-step regression and classification, scale-aware margin loss, a feature supervision module, and receptive field enhancement to improve small-face detection. It achieved a 99.11% true positive rate at 1,000 false positives on the FDDB dataset. MogFace [6], introduced in 2022, brought further innovations with its adaptive incremental anchor mining, selective scale enhancement, and context-aware modules. These enhancements enabled the model to surpass 90% mAP@50 on the WIDER FACE dataset, making it one of the top performers in the field.

Out-of-distribution (OOD) detection is a critical aspect of machine learning, particularly in multi-label classification tasks where models must handle unfamiliar data. Recent work introduces YolOOD, a novel method that integrates object detection concepts—specifically inspired by the YOLO (You Only Look Once) framework—for effective OOD detection in complex visual scenes [41]. Unlike traditional approaches that rely on external datasets, YolOOD uses bounding-box annotations to represent both in-distribution and OOD data. It also employs passive negative learning, leveraging unlabeled regions within training images to improve detection performance. An additional strength of YolOOD is the use of the objectness score from YOLO, which helps generalize the rejection of irrelevant objects. Moreover, the research incorporates large language models to automate dataset labeling and bounding box generation, enhancing scalability. Further, YolOOD addresses the largely underexplored domain of multi-label OOD detection and introduces new benchmark datasets to capture its complexity. Experimental results demonstrate that this study significantly outperforms existing OOD detection methods and standard YOLO models across various benchmarks. Notably, it reduces FPR95 by up to 9.53%, and when combined with JointEnergy, achieves improvements of up to 10.49% across multiple datasets. The model’s optimal performance is associated with a specific grid-cell configuration of (0.0, 0.1, 0.5). These findings underscore YolOOD’s potential for enhancing real-world applications such as autonomous systems and medical diagnostics by offering a more robust and scalable solution to OOD detection challenges.

Study [42] introduces the LUD-YOLO model for object detection using unmanned aerial vehicles (UAVs), with a focus on feature fusion and the reduction of model weights. The authors implement lightweight modifications through network slimming techniques, resulting in enhancements in both model size and runtime efficiency. The proposed model achieves notable improvements in detection accuracy across diverse scenarios and demonstrates effective target localization, although some performance limitations are observed in densely populated scenes. Ablation studies reveal that the reported detection performance decreases marginally by 0.1%–0.3% following pruning operations. Nevertheless, the model achieves a reduction in parameters by 7.35%–7.76% without a substantial compromise in detection performance. The integration of an Attention-based Feature Pyramid Network (AFPN) and Biform module contributes to a balanced performance, enabling the model to handle complex detection tasks with improved robustness and generalizability across various operational contexts.

The Segment Anything Model (SAM) [43] is introduced as a promptable image segmentation framework capable of generating valid segmentation masks in real time. Designed to address the inherent ambiguity in segmentation tasks, SAM is capable of producing multiple masks for a single prompt input. The model’s performance is comprehensively evaluated across 23 segmentation datasets, where it consistently demonstrates high-quality mask generation. Notably, SAM produces accurate segmentation masks from a single foreground point, closely approximating manually annotated ground truth data.

Study [44] explores the application of the You Only Look Once–Neural Architecture Search (YOLO-NAS) algorithm for object detection in the context of autonomous driving. To enhance detection precision, the authors integrate the Chimpanzee Leader Election Optimization (CLEO) algorithm with YOLO-NAS. CLEO operates by iteratively evaluating the fitness values of male and female chimpanzees to construct new candidate solutions, concluding the optimization process once a predefined criterion is met or the maximum number of iterations is reached. Furthermore, the YOLO-NAS model augmented with the CLEO optimizer reported substantially improved detection performance on the BDD100K dataset compared with the baseline YOLO-NAS model. The proposed method also reported improvements in recall and precision while balancing detection speed and accuracy. These enhancements underscore the efficacy of the CLEO optimizer in boosting real-time object detection performance for autonomous driving applications.

The study [45] proposes a Discriminative Deformable Part Model (DDPM) for pedestrian detection, which leverages machine learning to decompose human figures into deformable parts. This model introduces a feature transformation technique designed to effectively distinguish between occluded and non-occluded pedestrian features. A novel evaluation strategy is employed, utilizing additional visible bounding boxes during detection to improve localization accuracy.

To address the challenges posed by partial occlusions, the method incorporates dynamic part selection mechanisms, thereby reducing reliance on manual configuration. Although severe occlusions remain a challenge, the approach significantly enhances detection performance in moderately occluded scenarios. Experimental results demonstrate that the DDPM outperforms existing methods—including YOLOv11, HF-YOLO, EFF-YOLO, and MSCD-YOLO—in terms of detection performance on the Pascal VOC 2012 dataset. Furthermore, the proposed model exhibits strong performance on both the Pascal VOC and VisDrone-DET2019 Detection datasets, indicating its robustness across varying environments. The inclusion of a diverse pedestrian dataset enables comprehensive evaluation under different levels of occlusion. Precision-recall curve analysis further substantiates the superiority of the method compared to contemporary object detection networks, particularly in handling occluded pedestrian instances.

### A. Problem identification

The primary problem identified is that current deep learning models for face detection, while achieving high mAP@50 (above 80% mean average precision) on standard, controlled datasets, often fail in “the wild” due to variations in pose, direction, occlusions, and distance from the camera [2,3]. Publicly available datasets often feature posed images with faces directed towards the camera, which do not represent real-world conditions.

Single shot or YOLO-type detectors predict objects on fixed-resolution grid-based feature maps. In this setting, the object center assignments determine the grid cell responsible for predicting the bounding box. When several small objects are densely packed inside a grid cell, object centers may compete within the same or neighboring grid locations, which reduces localization mAP@50 and recall.

Patch-wise inference partially alleviates this problem by dividing a large image into smaller regions and applying the detector separately to each region. In this setting, the number of grid cells is effectively increased for small objects detection. However, using a fixed number of patches for every image increases computation and may magnify irrelevant small regions that produce false positives.

The unique contribution of this work is therefore an image adaptive patch selection mechanism with modified YOLOv5 architecture. We propose to use Cr channel color probability maps and fuzzy inference to estimate the number of patches required for each input image. Once the number of patches is determined, the modified YOLOv5 architecture with additional convolutional layers and detection heads is used for each patch for small face detection. This balances small face sensitivity against computational cost and false positive generation.

## Proposed methodology

Fig 1 illustrates our proposed framework for face detection. The framework begins with the creation of a probable face region map using color maps. These color maps facilitate the estimation of the appropriate patch size for face detection. Subsequently, the input is forwarded to a modified YOLOv5 architecture, optimized for small object detection, along with the calculated patch size to detect faces within the image.

**Fig 1:**
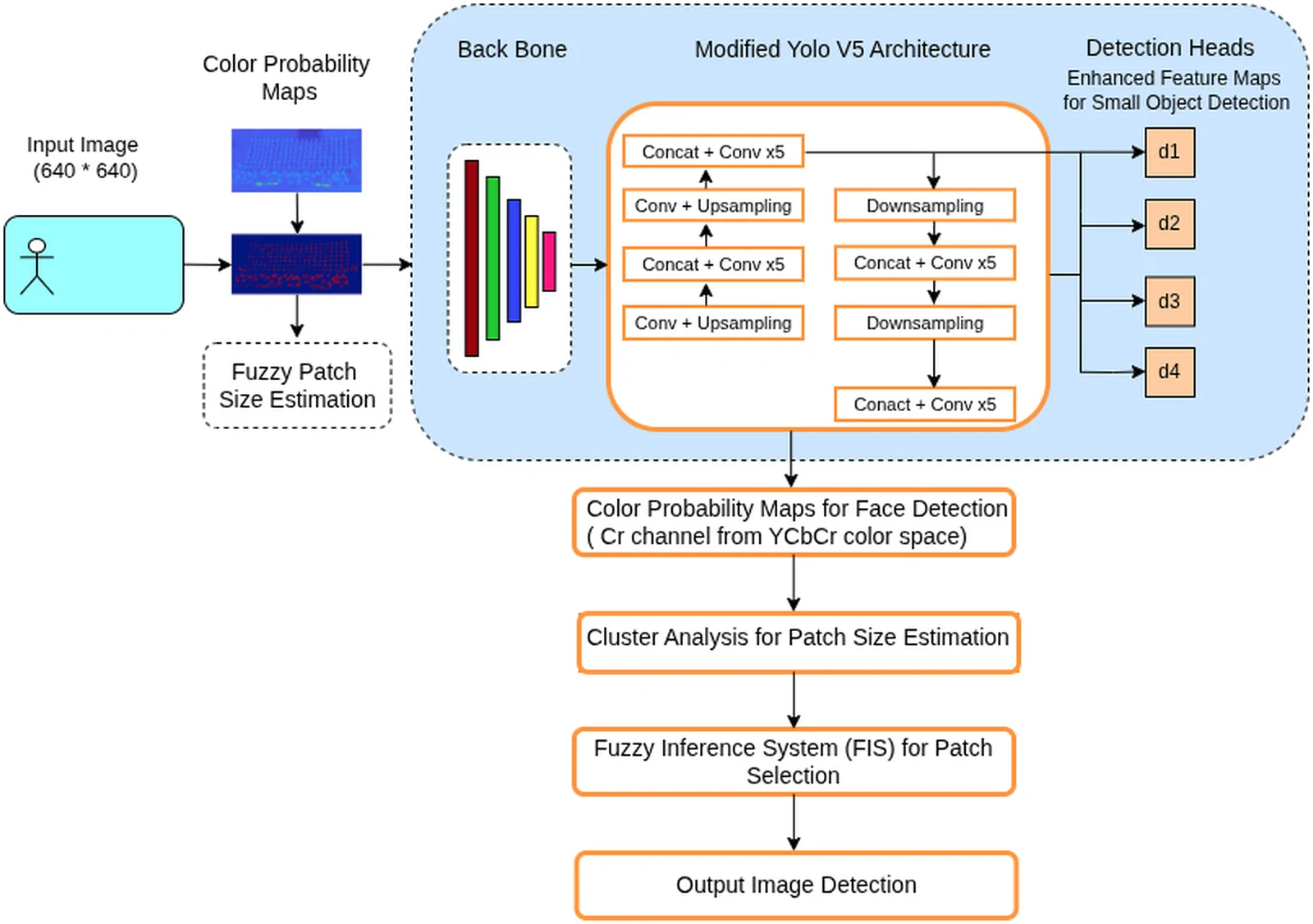
Block Diagram of Proposed Methodology.

YOLOv5 represents a family of strong object detectors. Almost all of the original detectors work on the principle of anchor boxes. Due to the inherent processing of this detection strategy, the detection of smaller objects becomes difficult compared with the image size. In the year 2021, Rehman et al. [46,47] proposed the use of patches in training and testing to reduce the severity of this problem. In the proposed method, we take this approach one step further by modifying the YOLOv5 backbone and neck layers and the inference-time detection process to facilitate small-object detection.

### A. Existing YOLOv5 architecture

YOLOv5 is a widely adopted, real-time object detection model known for its balance between mAP@50 and inference speed, making it effective for a broad range of computer vision tasks [48]. As part of the YOLO (You Only Look Once) family, YOLOv5 continues the tradition of single-shot detection introduced by the original YOLO framework [8]. Its multiple model variants accommodate different deployment needs and hardware constraints [48]. Moreover, the YOLOv5 framework, as shown in Fig 2, is structured into four essential components, each playing a pivotal role in the detection pipeline.

**Fig 2:**
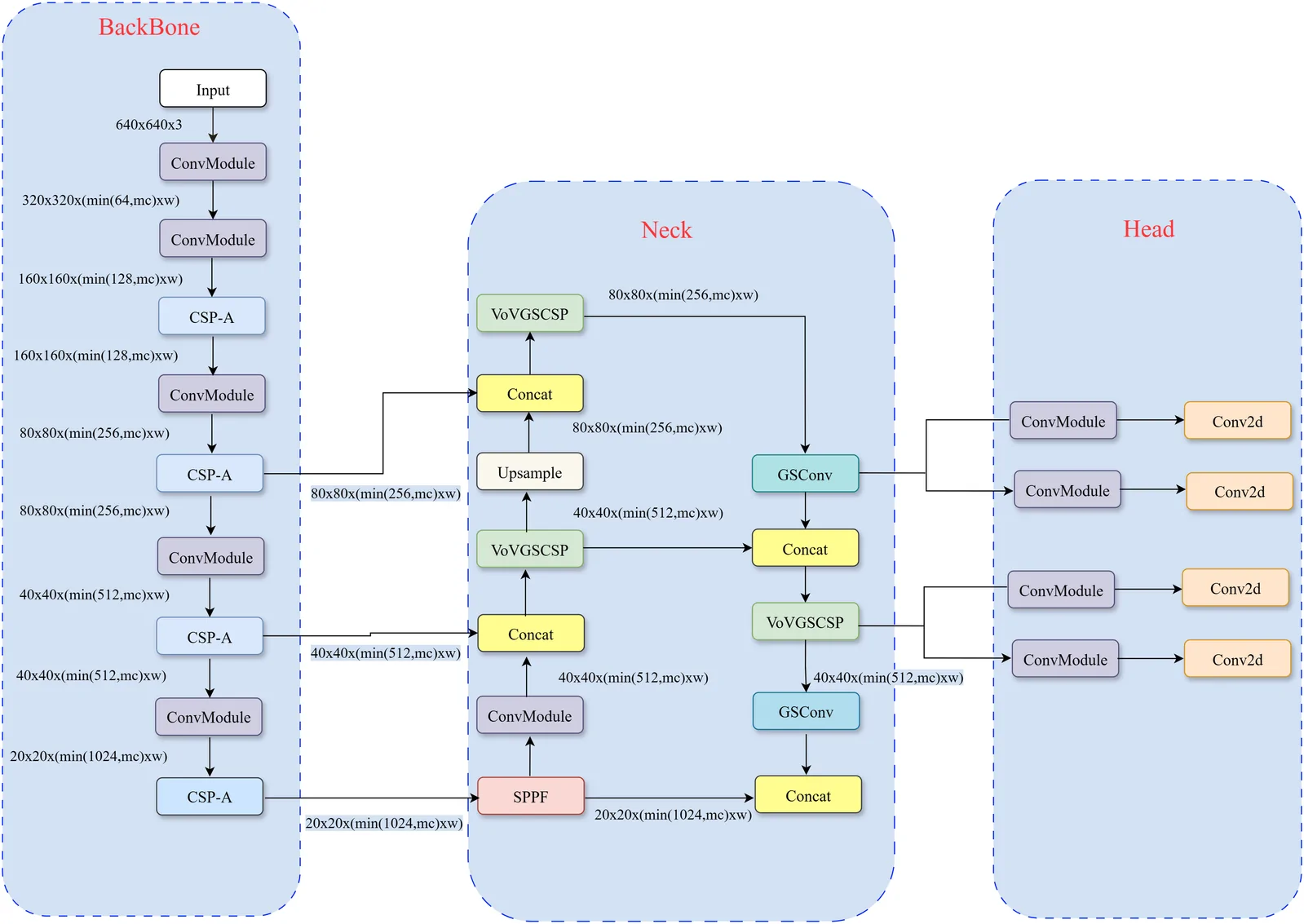
Existing Architecture of YOLOv5.

#### Input layer.

The input layer processes raw images and resizes them to a standard format, typically 640×640 pixels. It also includes preprocessing operations such as normalization, mosaic augmentation, and auto-padding to ensure consistent input across various image sources.

#### Backbone.

The backbone is responsible for feature extraction from the input image. YOLOv5 employs a Cross Stage Partial Network (CSPDarknet) as its backbone, which enhances gradient flow and reduces computational redundancy while maintaining mAP@50. This component captures low-level to high-level spatial information critical for object recognition [48,49].

#### Neck.

The Neck module is designed to generate multi-scale feature maps that help detect objects of varying sizes. YOLOv5 uses a combination of Feature Pyramid Network (FPN) and Path Aggregation Network (PANet) to strengthen the semantic representation of features at different scales. Additionally, the use of CSP (Cross Stage Partial) connections in the Neck improves feature reuse and reduces computational load, leading to better generalization [48–51].

#### Head (prediction layer).

The prediction head comprises detection layers at three different scales, allowing the model to detect small, medium, and large objects simultaneously. Each head predicts the class, bounding box coordinates, and objectness score for potential objects within the image [48].

YOLOv5 introduces several advancements that distinguish it from earlier versions and competing models [48].

#### Focus Module.

This module slices the input image into patches before feeding it into the network, effectively reducing the spatial resolution while preserving critical features. It accelerates computation by minimizing the number of operations in the early layers.

#### CSP connections.

The integration of Cross Stage Partial (CSP) connections in both the backbone and neck enables improved feature learning and gradient flow, thereby enhancing the model’s performance and stability.

#### Anchor box optimization.

YOLOv5 leverages K-means clustering and genetic algorithms to optimize anchor boxes for each dataset automatically, improving localization mAP@50 and model adaptability across different domains.

#### Dynamic architecture and augmentation.

YOLOv5 utilizes techniques such as mosaic augmentation, label smoothing, and adaptive image scaling to enhance generalization. The architecture is modular and scalable, allowing for easy deployment on various devices and environments.

#### Scalability via model variants.

YOLOv5 is available in multiple variants, including YOLOv5n (nano), YOLOv5s (small), YOLOv5m (medium), YOLOv5l (large), and YOLOv5x (extra-large), each tailored to different performance and resource requirements. These variants differ in depth and width multipliers, allowing users to choose the best model for their specific application and hardware.

As shown in Fig 3, the existing YOLOv5 block diagram is composed of four main components, Input, Backbone, Neck, and Head, each playing a vital role in object detection. The process begins with an input image, typically resized to 640×640×3 to maintain consistency in dimensions and channels. This image is then passed through the Backbone, which in YOLOv5 is CSPDarknet, a variant of the Darknet architecture enhanced with Cross Stage Partial (CSP) connections to improve gradient flow, reduce computation, and enhance feature learning. The extracted features are forwarded to the Neck, which consists of a Feature Pyramid Network (FPN) and a Path Aggregation Network (PANet). The FPN facilitates the combination of semantic-rich low-resolution features with high-resolution features, while the PANet strengthens localization capabilities through bottom-up path augmentation, enhancing the model’s ability to detect objects at multiple scales. Finally, the refined features are sent to the Head, which comprises detection layers operating at three different scales. This multi-scale detection mechanism enables YOLOv5 to effectively identify small, medium, and large objects within the image by predicting bounding boxes, object confidence scores, and class probabilities. This streamlined yet powerful design allows YOLOv5 to achieve both speed and mAP@50 in real-time object detection tasks.

**Fig 3:**
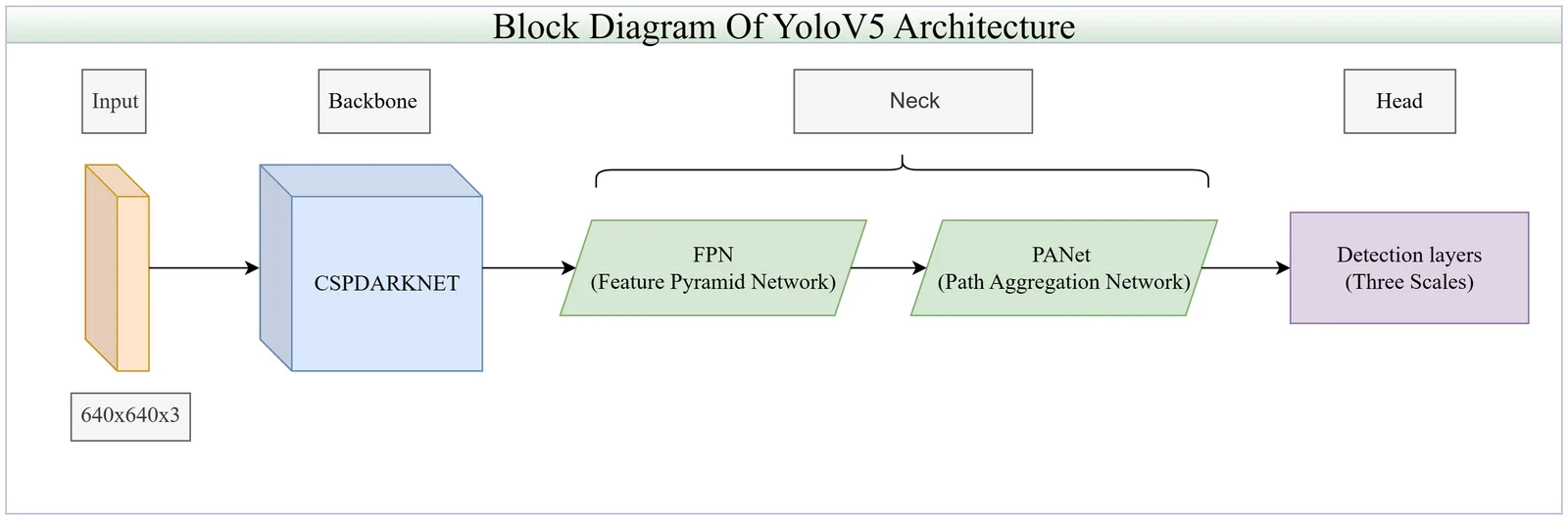
Block Diagram of YOLOv5.

### B. Modifications in YOLOv5 backbone and neck layers

We now discuss why the default YOLOv5 architecture was modified, what changes were made, and how these changes support the detection of small objects.

#### i. Why are modifications necessary?.

One thing we want to emphasize is that YOLOv5 has been designed by keeping in view the high performance for object detection on the datasets like COCO, ImageNet, etc. These datasets usually have big object sizes as compared to the image size. Therefore, for the detection of smaller objects, i.e., those objects that have size ratio less than 5% of the image size, redefining the object detector is necessary.

The first challenge arises from the feature maps passed through the neck and detection heads of the default YOLOv5 model. There are a total of three detection heads in the default YOLOv5 model. Each detection head is responsible for detecting objects of a specific size–-small, medium, or large–-relative to the input image. The detection head responsible for the detection of small-sized objects receives the feature map that traverses from the backbone to the neck and then to the detection head. The size of this feature map is only 80x80 dimensions.

In addition, the head responsible for the detection of medium-sized objects receives a 40x40 feature map. This feature map also traverses from the backbone to the neck and then to the detection head. Finally, the head responsible for detecting large objects relative to the input image receives a 20x20 feature map. This feature map is directly taken from Spatial Pyramid Pooling Fast (SPPF) layer and traverses through the neck and then reaches the detection head. All the calculations are done with the input image size of 640x640 pixels.

We analyze that the feature maps taken from the backbone and concatenated with the neck and provided to the detection head are very small. These feature maps work well for bigger objects with respect to the image size, however, these limit the detection of small-sized objects. For small object detection, we need bigger feature maps. Therefore, modifications in the default YOLOv5 network model were deemed necessary for the detection of small-sized objects such as faces.

#### ii. What are the modifications?.

The key modification is the use of larger feature maps. We solve the issue of small object detection by extracting feature maps (shown in Fig 4) from the initial layers of the backbone that provide larger feature maps. Furthermore, to support these feature maps we modified the neck and added four convolutional blocks that supported the adjustment of features extracted from the initial layers of the backbone in the deep network model. In addition, we also added one additional detection head for small-object detection.

**Fig 4:**
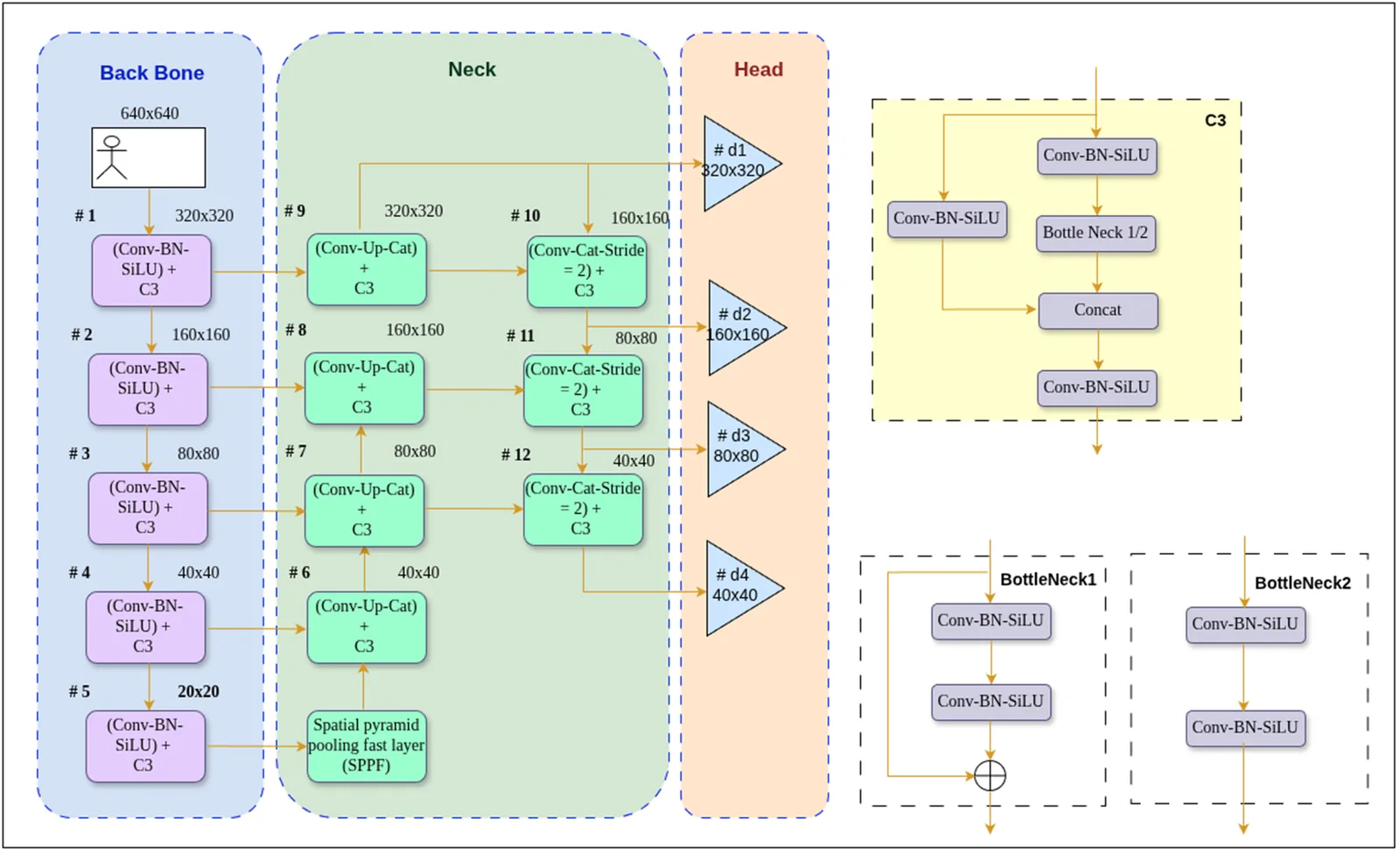
The proposed modified architecture of YOLOv5. It shows the modification in backbone, neck, and head are focused to increase the model sensitivity towards the detection of small objects.

#### iii. How are the modifications made?.

As illustrated in Fig 4, the proposed method modifies the YOLOv5 framework to accommodate a larger feature map. To achieve this, four convolutional layers (C3) and two detection heads were added. One C3 layer was integrated into the backbone, while the remaining three were added to the neck of the architecture, illustrated in comparison with YOLOv5 architecture in Table 1. Furthermore, we will provide a detailed discussion of these modifications within the default YOLOv5 network, considering an input image size of 640x640 pixels. It is worth noting that low-resolution faces can pose a challenge in face detection tasks [19].

**Table 1:** Comparison of the original YOLOv5 and the proposed YOLO-SOD architecture.

Component	Original YOLOv5	Proposed YOLO-SOD
Detection heads	3	4
Detection levels	P3, P4, P5	P1, P2, P3, P4
Detection strides	8, 16, 32	2, 4, 8, 16
Feature maps at 640×640 input	80×80, 40×40, 20×20	320×320, 160×160, 80×80, 40×40
320×320 head	Absent	Added
160×160 head	Absent	Added
20×20 head	Present	Removed
Added C3 blocks	–	4 additional C3 blocks
Main purpose	General object detection	Better small-face detection

The C3 (#1) layer that has been added in the backbone provides feature maps of 320x320 dimensions. This occurs because the input image features are downsampled with a stride of two inside the CBS block (convolution, batch normalization, and sigmoid linear activation function). We took these features and connected them to another C3 (#9) layer inside the neck. This way, these features can be directly provided to the small-object detection head (#d1) that is connected to the neck layer (#9) directly.

Furthermore, we added another C3 layer (#8) within the neck to manage the 160x160 feature maps. Feature maps from C3 layer (#2) are connected with the C3 layer (#8). These feature maps have dimensions of 160x160. Detection head (#d2) is connected with the C3 layer (#8) inside the neck layer to further accommodate the detection of small-sized objects. Feature maps are given to the detection head (#d2) through another added C3 layer (#10). This head (#d2) processes and finds the objects on the bigger feature maps as compared to the default network.

The remaining heads (#d3 and #d4) were kept intact with the network. These heads (#d3 and #d4) receive feature maps of sizes 80x80 and 40x40 from the neck layers. However, the detection head that accommodates smaller feature maps (20x20 dimension) for large objects has been removed. The rationale behind removing this head is that the bigger objects inside images could be sufficiently accommodated by the detection head (#d4) that processes the feature maps of 40x40 dimension.

### C. Cr-channel color probability maps for face detection

The modified YOLOv5 architecture can be used to detect small objects such as faces in images. When combined with the patching method [47], it can effectively detect both near- and far-distance faces when the patch size is known before detection for that particular image. We needed to selectively change the patch size for far away faces (very small objects) and near faces (big objects) to obtain better detection results. We observed this during experimentation with WIDER FACE dataset. We also observed that when the size of the patch is smaller for near faces (large objects), multiple detection bounding boxes appeared around the face and the network was not able to predict the final bounding box for near faces. On the other hand, when the size of the patch is larger for far faces (small objects), the detector started to miss the true positives. Thus, the size selection of patch resulted in false negatives and false positives.

We investigate the usefulness of color information cues for adaptive patch selection. We sampled 500 images from the WIDER FACE training set and cropped those images in such a way that only one human is shown inside a single cropped window. We then resized all the cropped windows to windows with a spatial resolution of 100×41 pixels and calculated the average channel response heatmaps for RGB, HSV, and YCbCr color spaces.

Experiments show that the YCbCr color space provides useful clues about the human skin tone. This is because the YCbCr color space separates luminance (or brightness) in the Y channel from the blue-difference and red-difference chrominance components in the Cb and Cr channels, respectively. Fig 5 shows the intra-class correlation maps on RGB, HSV, and YCbCr color spaces. The red spot around the face region in the Cr channel of Fig 5(c) shows the concentrated response around visible skin regions, including the face and other exposed skin areas.

**Fig 5:**
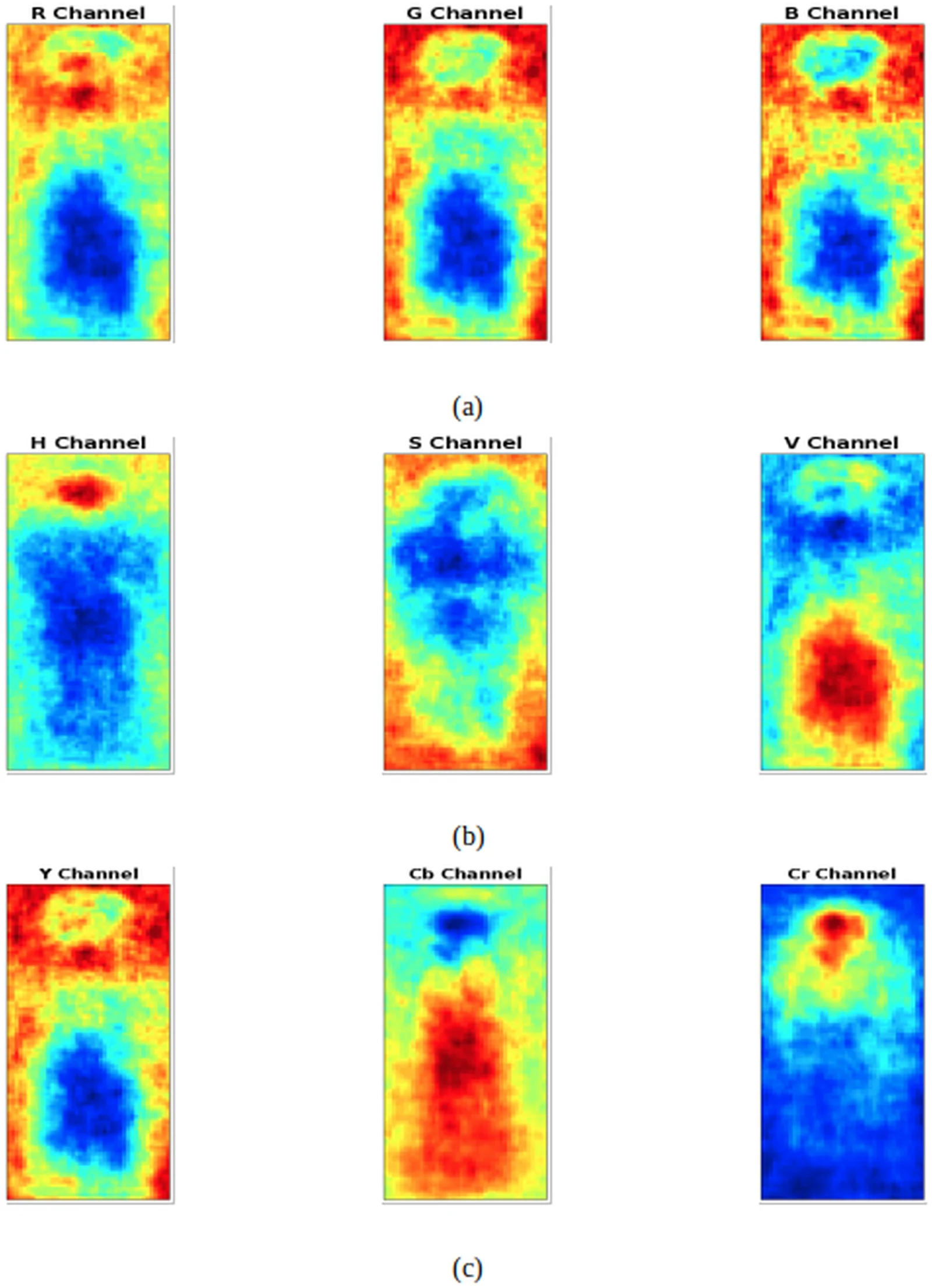
Intra-class correlation heat maps for analyzing the face location. Red areas represent higher correlation areas in the colorspace channels (a) RGB colorspace, (b) HSV colorspace, and (c) YCbCr color space.

Therefore, we propose to use the Cr channel as a heuristic scene level cue for the estimation of number of patches on that particular image rather than a direct face detector. We learn the Cr values using the smoothed face and background Cr histograms from the training data. The histogram values are then converted into likelihood ratios to produce pixel level face color probability maps. The resulting color probability maps were thresholded to obtain Cr response clusters. The number and density of those clusters were used as scene level features to estimate the required number of patches’N’. Therefore, the learned Cr model controls the image partitioning process rather than directly detecting faces.

For patch localization on images, the Cr color probability map was fused with a weak YOLOv5 heatmap, i.e., heatmap of YOLOv5 detections whose confidence values are in the range (0.001, 0.2). The fusion weight values of 0.65 and 0.35 were used for fusing Cr color probability map and YOLOv5 weak detection heatmap, respectively, to identify candidate patch centers.

The image would then be divided into the estimated’N’ number of patches and the patches are then given to the proposed detector for face detection.

To quantitatively support the heatmap observations, we computed a head region contrast score (HCS) for each channel using the average maps generated from 500 aligned WIDER FACE crops resized to 100×41 pixels. A fixed central head region of interest was defined using rows 5–29 and columns 8–32. The HCS measures the normalized difference between the mean response inside the head region and the mean response in the remaining part of the crop:


$$\begin{array}{c} {HCS}_{c}=\frac{\left|\mu_{head,c}-\mu_{non\text{-}head,c}\right|}{\sigma_{full,c}} \end{array}$$ (1)

where $\mu_{head,c}$ is the mean response of channel *c* inside the predefined head region, $\mu_{non\text{-}head,c}$ is the corresponding mean response outside this region, and $\sigma_{full,c}$ is the standard deviation of the complete channel map. Normalization by the full-map standard deviation enables a fair comparison between channels having different numerical ranges. A higher HCS indicates stronger separation between the head regions and the remainder of the images.

As reported in Table 2, the Cr channel achieved the highest HCS of 1.818, exceeding the Cb and R channels by approximately 12.2% and 47.8%, respectively. This result quantitatively supports the heatmap observation that the Cr channel provides the strongest single channel separation of the predefined head region in the aligned crops.

**Table 2:** Head region contrast scores for the evaluated RGB, HSV, and YCbCr channels. A higher score indicates stronger separation of the head region from the remaining image area.

Color space	Channel	HCS
RGB	R	1.230
	G	0.508
	B	0.208
HSV	H	1.111
	S	0.731
	V	1.018
YCbCr	Y	0.740
	Cb	1.620
	**Cr**	**1.818**

It should be noted that the WIDER FACE dataset does not provide explicit annotations for skin tone, illumination conditions, or camera characteristics. Therefore, the present analysis does not establish that Cr channel responses are invariant across these factors.

Now, Fig 6 shows the color maps of images. In Fig 6, column (a) shows the color map obtained from the Cr channel, and column (b) represents human face and skin identifications using thresholding on the images from column (a).

**Fig 6:**
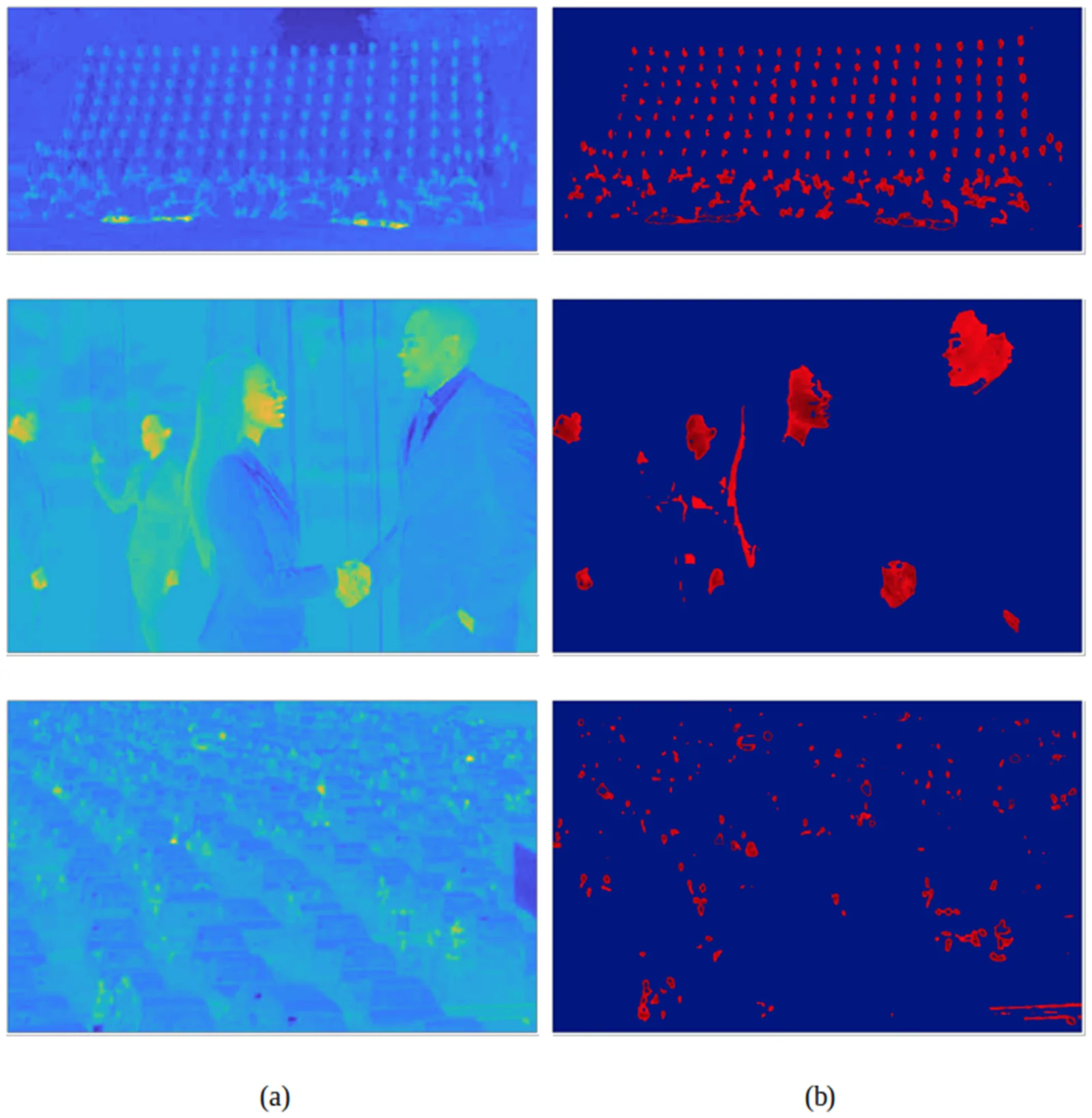
Color maps using Cr channel of YCbCr color space for face detection. (a) Cr channel color maps (b) Cr color maps using threshold for visualization purposes.

Once color maps are obtained, we find the locations and densities of clusters of pixels in the Cr channel. We hypothesize that each cluster may provide a coarse estimate of a skin-related candidate in an image. We determine the number of clusters using the pixel connectivity method. After determining the number of clusters, we find the density for each cluster in the Cr channel color map by counting the number of pixels associated with each cluster. By using the count and density values of clusters inside the Cr channel color map, we obtain a coarse cue for selecting patch sizes that are suitable for scenes that contain near- and far-distance faces. Although the number and density of clusters do not provide the actual number of faces or their distances from the camera, we use this information cue to estimate the patch sizes needed to divide the input image into sub-images for the detection of small and large faces.

We again hypothesize that a high number of low density clusters may indicate a scene in which smaller patches are more suitable for detecting small or possible distant faces. On the other hand, a low number of high density clusters may indicate that larger patches are more suitable for detecting larger or nearer faces. Due to this reason, we refer to these Cr-channel maps as color probability maps. These maps give an indication about the size of the patch for small object detection. Further, we use this information about the number and density of clusters with the Fuzzy Inference System to determine the final size of the patch to be used for face detection in the input image.

**Quantitative validation of Cr-cluster count and scale cues.** To investigate the Cr channel cues used by the fuzzy inference system, image and face level analyses were performed on 136 WIDER FACE training images containing 1,310 annotated faces.

At the image level, the number of Cr candidate clusters was compared with the number of annotated faces. As summarized in Table 3, images containing more faces generally produced more Cr candidate clusters. Images with low, medium, and high face counts produced averages of 20.18, 30.59, and 72.95 clusters, respectively. Moreover, 78.8% of the annotated faces had at least one Cr candidate cluster within the evaluated face region. Evaluated face regions were calculated by enlarging the original ground truth annotations by 1.5 times. These results support the use of image level cluster count as a coarse indication or cue of the relative abundance of candidate skin related regions. This does not imply that every cluster corresponds to an actual face.

**Table 3:** Image level relationship between annotated face abundance and Cr candidate clusters.

Face-count group	Images	Mean faces	Mean Cr clusters
Low (≤2 faces)	61	1.54	20.18
Medium (3–6 faces)	32	4.44	30.59
High (> 6 faces)	43	24.98	72.95

For the investigation of the cluster size cue, the relative scale of each annotated face was calculated from its original bounding box height with respect to the corresponding image height as follows.


$$\begin{array}{c} r_{j}=\frac{h_{j}^{face}}{H_{j}^{image}} \end{array}$$ (2)

where $h_{j}^{face}$ is the height of the *j*th face bounding box and $H_{j}^{image}$ is the height of the corresponding image. The normalized face scales were divided into approximately equal small-, medium-, and large-scale groups as follows.


$$\begin{array}{cc} \text{Small scale: } & r_{j}\leq 0.022398\\ \text{Medium scale: } & 0.022398<r_{j}\leq 0.048316\\ \text{Large scale: } & r_{j}>0.048316 \end{array}$$ (3)

It means that for an image with a height of 1000 pixels, these thresholds represent the faces as small for heights up to 22 pixels, medium for heights in between 22–48 pixels, and large for heights more than 48 pixels. These categories represent relative face scale and do not represent physical distance from the camera.

As reported in Table 4, the mean Cr cluster size increased from 46.78 pixels for small faces to 209.83 pixels for medium faces and 5012.90 pixels for large faces. The median cluster size has also shown the same trend. These results indicate that larger face instances generally produce larger Cr candidate regions, which supports the use of cluster size or density as a coarse face scale cue.

**Table 4:** Cr channel cluster size statistics for different normalized face scale groups.

Face scale	Faces	Mean cluster size	Median cluster size
Small	438	46.78	43.5
Medium	436	209.83	126.0
Large	436	5012.90	1199.5

The two Cr channel measurements, i.e., cluster count and density, therefore have separate roles in the proposed method. Image level cluster count provides a coarse indication of the relative abundance of candidate regions, whereas cluster size or density provides a cue about their spatial scale. The fuzzy inference system combines these cues to select the number and size of image patches. Neither measurement is used to directly determine the exact number of faces or their physical distance from the camera.

It should be noted that the cluster count is measured at the image level and it is only used as a coarse indication of the relative abundance of candidate skin related regions. Furthermore, cluster density describes the number of pixels that are associated with each candidate region and it is used as a coarse scale cue. Therefore, cluster count and density are used jointly to select the patch configuration and not to directly estimate the number or physical distance of faces.

### D. Fuzzy patches for near and far face detection

A conventional threshold based strategy would require several manually selected decision ranges for the cluster count and cluster density. These threshold ranges would need extensive tuning on the training set. On the other hand, training an additional deep network to predict the appropriate number of patches for every input image would introduce extra model complexity and supervision solely for scale selection.

We therefore adopt a lightweight fuzzy inference system because it can combine a small number of variables, namely cluster count and cluster density, and can provide flexible decisions through overlapping membership functions rather than rigid thresholds. This design enables patch number selection very simply using the written rules without requiring an additional learned model.

Mamdani inference has been used for this application. As shown in Fig 7, rules were decided by looking through the lens of color probability maps for near and far face detection system. It means that a high number of low-density clusters is treated as a cue for selecting smaller patches, which are more suitable for detecting small or distant faces. And a low number of high-density clusters is treated as a cue for selecting larger patches, which are more suitable for detecting larger or nearer faces.

**Fig 7:**
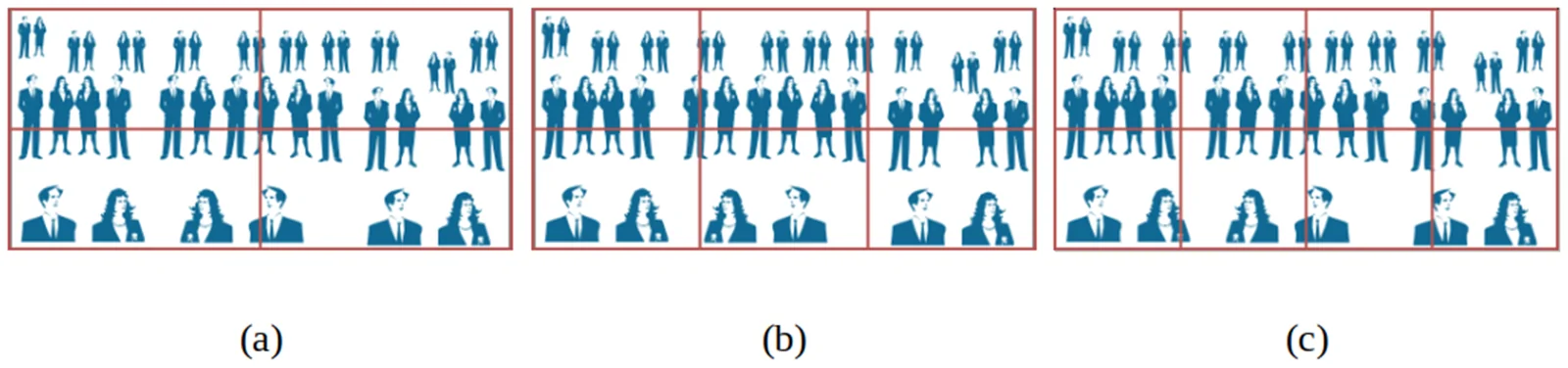
Selection of number of patches for small object detection (a) four patches (large size patches), (b) six patches (medium size patches), and (c) eight patches (small size patches).

Hence, large patches can be used for near objects to avoid false positives, whereas small patches can be used for far objects to reduce missed true positives. Based on the obtained patch size we divide the image into’N’ number of sub-images for the proposed detector. The fuzzy rules used in the proposed inference system are listed below.

1. If the number of clusters is high and density of clusters is low then the size of the patches is small.
2. If the number of clusters is low and density of clusters is high then the size of the patches is large.
3. If the number of clusters is medium and density of clusters is medium then the size of the patches is medium.
4. If the number of clusters is high and density of clusters is high then the size of the patches is medium.
5. If the number of clusters is low and density of clusters is low then the size of the patches is small.

Rules 1 and 2 address far- and near-face detection, respectively. Rules 3 and 4 are used when the color probability map distribution favors a medium patch configuration. Rule 5 is used when the observed cluster distribution favors smaller patches for potentially sparse and small face instances inside an image.

As shown in Fig 8, a pipeline is designed to determine optimal patch sizes for object detection using a modified YOLOv5 architecture guided by a fuzzy inference system. The process begins with a 640×640 input image, which undergoes transformation into a Cr channel color map, isolating chrominance information from the image. A thresholding operation is then applied to highlight areas of interest, producing a thresholded Cr color map. This threshold map is analyzed for cluster detection using a pixel connectivity map, which identifies the spatial distribution and density of object groupings in the image. These clustering results are then interpreted using fuzzy inference system rules: if the number of clusters is high and their density is low, smaller patches are chosen to capture sparse but numerous objects; if the number and density of clusters are both high, medium patches are selected; and medium density and cluster count also lead to medium patch sizes. Quantitative specifications of the fuzzy inference system are listed in Table 5

**Table 5:** Quantitative specification of the fuzzy inference system used for adaptive patch selection.

Input variable	Membership ranges	Membership function	Output patch sizes	Defuzzification method
Cr cluster count *C*	Low: 13.14–207.61; Medium: 29.04–213.73; High: 161.62–268.49 clusters	Gaussian	Small: 8; Medium: 6; Large: 4	Sugeno weighted average; final output assigned to the nearest patch number in {4,6,8}
Cr cluster density *D*	Low: 4161.32–15059.57; Medium: 7606.66–39360.93; High: 38226.00–48497.58	Gaussian	Small: 8; Medium: 6; Large: 4	Sugeno weighted average; final output assigned to the nearest patch number in {4,6,8}

**Fig 8:**
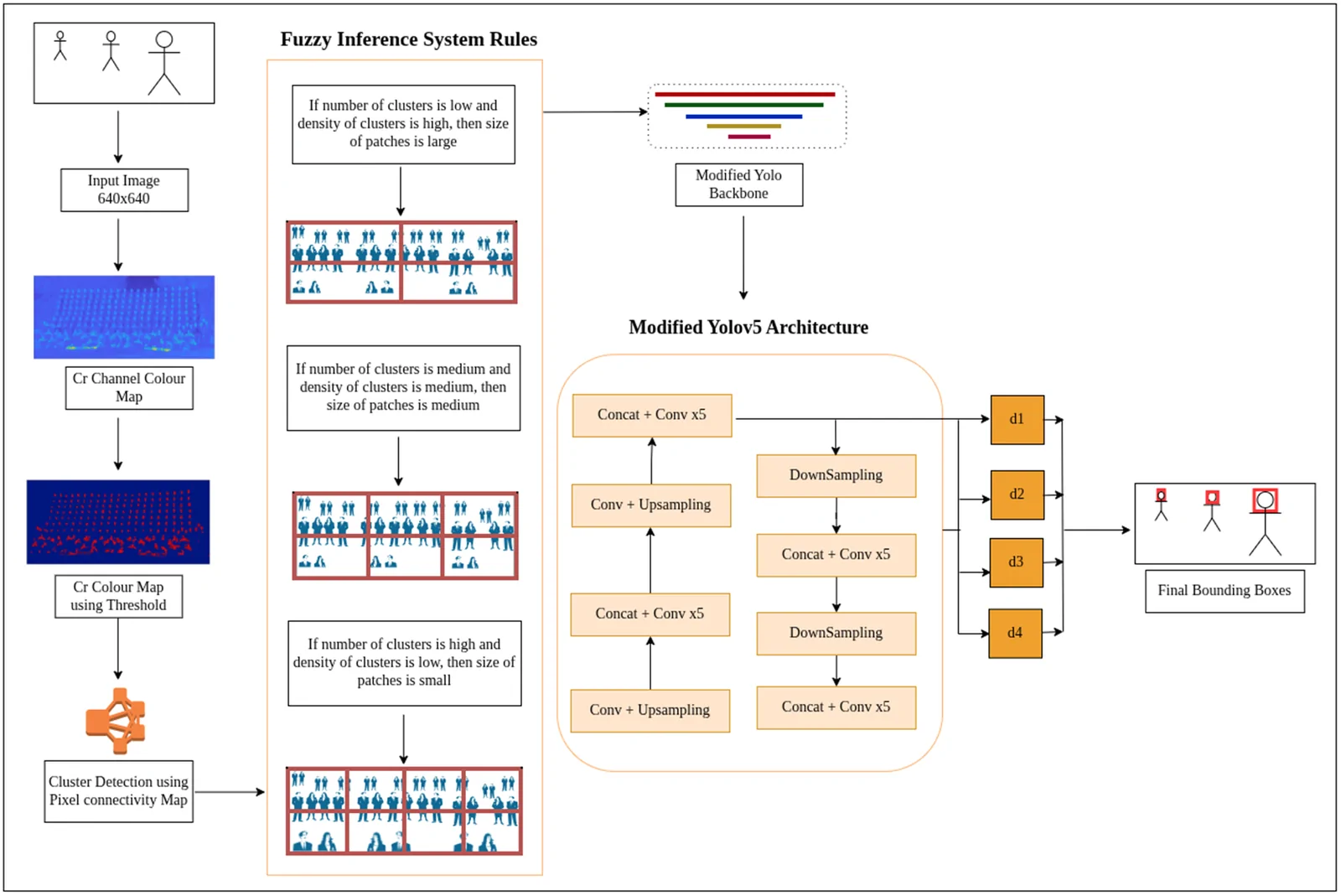
Fuzzy Cluster-Guided Patch Sizing for Multi-Scale Object Detection in YOLOv5.

Based on these fuzzy rules, patch size decisions influence how the image is processed by the modified YOLOv5 backbone, which feeds into a modified YOLOv5 architecture featuring multiple layers of convolution, upsampling, downsampling, and concatenation for multiscale feature learning. Four different detection layers (d1 to d4) process the features to predict objects at multiple scales. It should be noted that faces in the full image and cropped patches differ in scale and appearance distribution, therefore, a parallel detector has been trained on faces extracted using patches on the training images. This second detector works whenever the fuzzy rules are activated and patches are produced. We trained this detector to first learn from the full dataset and then perform transfer learning to learn how the faces look inside patches. This is because patches present faces after cropping and resizing. A normal face occupies a larger relative area within a patch.

Finally, the proposed detector predicts the bounding boxes after processing the image using the adaptively selected patch configuration. This cluster aware modification aims to improve detection mAP@50 in scenes with varying object distributions. The pseudocode of the proposed method is presented in Algorithm 1. [Codes and local dataset can be accessed from https://github.com/YawarGuguma/color-probability-maps]

**Algorithm 1 Face detection using color probability maps and a fuzzy-based framework**

Following is the description of variables:

*I*: Input image

*G*: Convolutional Block

*F*: Feature map

*d*: Detection head

$\bar{\rho }$: Average density of color pixel

1. $I\in {\mathbb{R}}^{640\times 640\times 3}$                       ← Input image

2. $Y_{CbCr}=YCbCr(I),\;\mathrm{split}(Y_{CbCr})=\{Y,C_{b},C_{r}\}$      ← Color space conversion

3. $M=\mathrm{threshold}(C_{r}),\;M\in \{0,1{\}}^{640\times 640}$    ← Binary mask from $C_{r}$ channel

4. $C=\{C_{1},C_{2},...,C_{n}\}$       ← Connected components

$n=|C|,\;P_{i}=\text{pixel-count}(C_{i})$

5. Color-probability-maps:

$\begin{array}{cc} \text{Clusters} & =C,\\ \text{Number of clusters} & =n,\\ \text{Density} & =(P_{1},P_{2},...,P_{n}) \end{array}$

6. Let number of clusters: *x*_1_ = *n*              ← Number of regions

7. Let $x_{2}=\bar{\rho }=\frac{1}{n}\sum_{i=1}^{n}P_{i}$    ← Average density of pixels

8. Fuzzy Rules:                ← Determines patch size based on *x*_1_ and *x*_2_

• *R*_1_: $x_{1}\in high$ and $x_{2}\in low\Rightarrow \text{patch-size}=small$

• *R*_2_: $x_{1}\in low$ and $x_{2}\in high\Rightarrow \text{patch-size}=large$

• *R*_3_: $x_{1}\in medium$ and $x_{2}\in medium\Rightarrow \text{patch-size}=medium$

• *R*_4_: $x_{1}\in high$ and $x_{2}\in high\Rightarrow \text{patch-size}=medium$

• *R*_5_: $x_{1}\in low$ and $x_{2}\in low\Rightarrow \text{patch-size}=small$

9. $\text{patch-size}=FIS(x_{1},x_{2})$ ← Fuzzy estimate of patch size

10. Patches=split(I,patch-size) ← Image patches based on fuzzy output

11. For each patch $P_{j}\in Patches$:

$\begin{array}{cc} C_{1}(P_{j}) & =F_{1}\in {\mathbb{R}}^{320\times 320\times C_{1}}\leftarrow \text{ Backbone modifications}\\ F_{2} & =C_{2}(F_{1}in{\mathbb{R}}^{160\times 160\times C_{2}}\leftarrow \text{ Neck modifications}\\ F_{3} & =C_{9}(F_{g})\\ F_{4} & =C_{1}(C_{8}(F_{2})) \end{array}$

$\begin{array}{cc} D_{1} & =d_{1}(F_{3}),\;D_{1}\in {\mathbb{R}}^{320\times 320\times k}\\ D_{2} & =d_{2}(F_{4}),\;D_{2}\in {\mathbb{R}}^{160\times 160\times k}\\ D_{3} & =d_{3}(F_{1}),\;D_{3}\in {\mathbb{R}}^{80\times 80\times k}\\ D_{4} & =d_{4}(F),\;D_{4}\in {\mathbb{R}}^{40\times 40\times k} \end{array}$

Where *k* is the number of anchor boxes × (classes + bounding box coordinates)

12. $YoloSOD(P_{j})=\{D_{1},D_{2},D_{3},D_{4}\}$         ← Output of model for each patch

13. Final detections =merge(YoloSOD(Patches))    ← Final face detection output

### E. Proposed dataset highlighting local attire conditions

The proposed local dataset emphasizes the challenges posed by pedestrians wearing traditional Eastern attire and local headgear, which often obscure facial features and pose significant hurdles for object detection models. This subset serves to rigorously evaluate the robustness of the proposed detector under real world conditions, where conventional state-of-the-art networks often exhibit degraded performance. Fig 9 shows the distribution of the face bounding boxes in the proposed dataset. The distribution shows that from the fixed perspective, faces recur at similar spatial locations.

**Fig 9:**
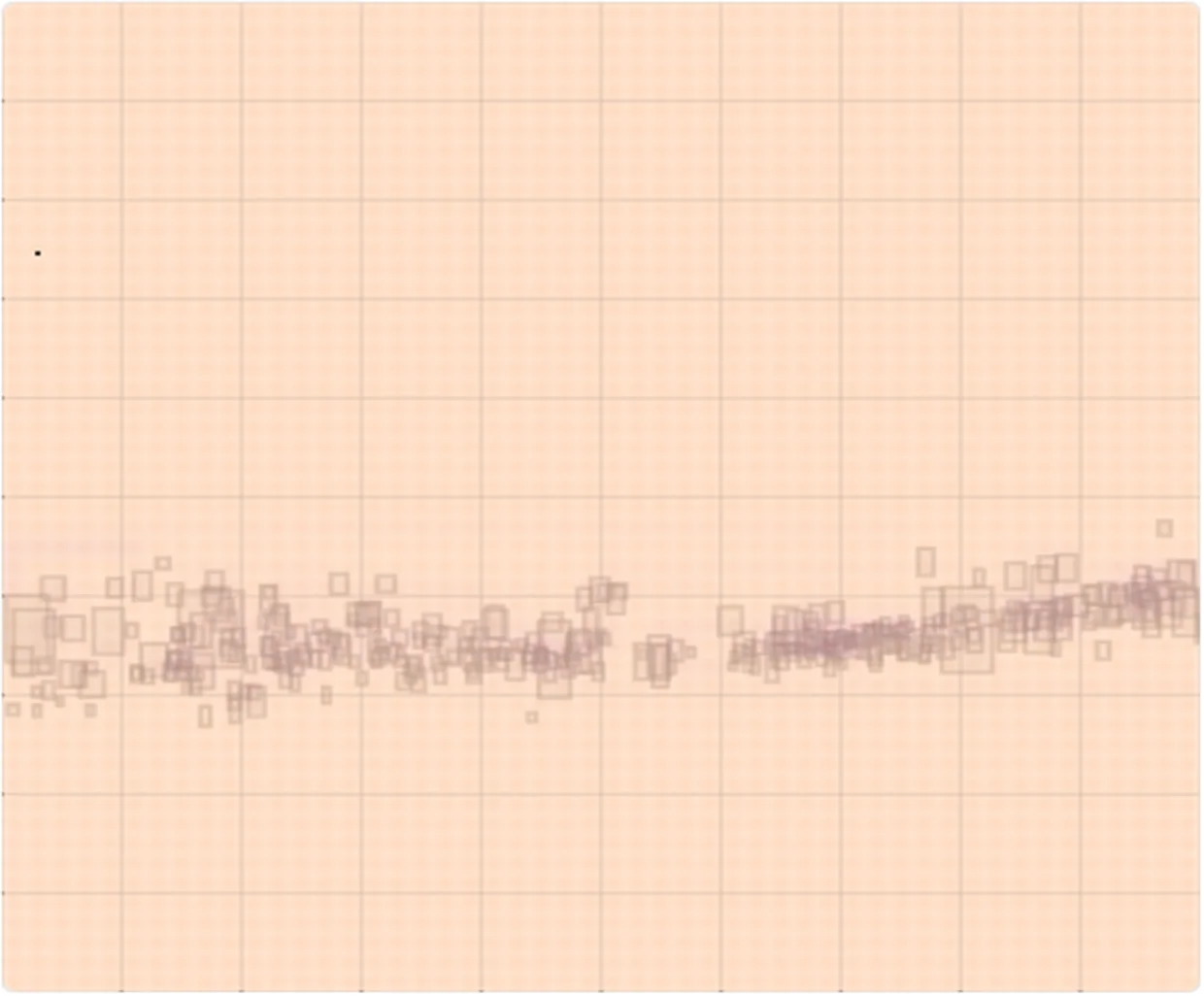
Bounding box distribution of faces in the proposed local dataset.

Table 6 shows the image and annotation distribution details of the proposed local dataset. Dataset was collected using a smartphone mounted on the dashcam of a car inside NED University. The dataset used here is a subset of a larger dataset that has been used in other research works and purposes. Annotation of the dataset was conducted using the open-source online platform Roboflow by utilizing its comprehensive suite of tools. The dataset comprises a total of 436 images, each with a resolution of 1920 × 1080 pixels, specifically curated to reflect urban environments. A total of 350 face annotations were made, yielding an average of approximately 0.8 annotations per image. Notably, 271 images contained no-face annotations, 83 images included a single face annotation, 67 images featured between two to four annotations, 12 images contained five to seven, and 3 images had eight to ten face annotations as shown in Table 7. No-face images were intentionally included by the authors to replicate the real situation. Faces are not always present in road scenes; therefore, these images help assess detector reliability when no faces are visible, particularly when the camera is fixed on the dashboard of a car. This also shows the authors’ intent to use and implement this project’s findings on an edge device.

**Table 6:** Image and annotation distribution details for the proposed local dataset.

Description	Results
Total images in local dataset	436
Total number of local annotations	350
Total number of images containing faces	165
Annotations per image	0.8
Image size	1920 x 1080

**Table 7:** Distribution of images and face annotations in the proposed dataset.

Faces per image	Number of images	Face annotations
0	271	0
1	83	83
2	42	84
3	11	33
4	14	56
5	6	30
6	5	30
7	1	7
8	1	8
9	1	9
10	1	10
**Total**	**436**	**350**

The proposed dataset includes a wide range of Eastern headgear and clothing styles. Furthermore, Fig 10 presents a few samples from the dataset that were cropped from the original local dataset images. These samples were cropped from the full image to emphasize the faraway faces from the camera. Moreover, the dataset contains multiple variations in headgear style that affect detector efficiency and enable evaluation of face detection in the wild.

**Fig 10:**
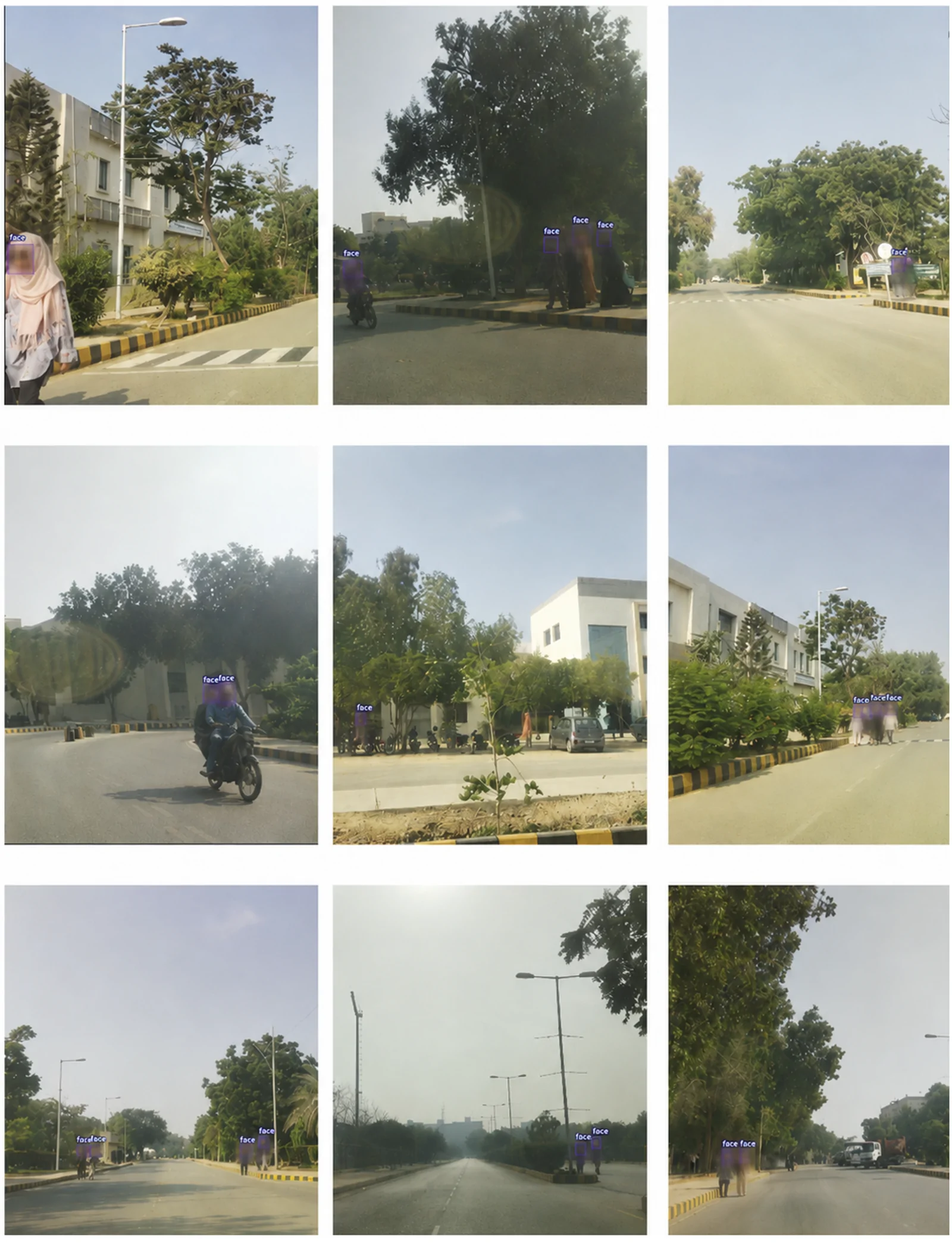
Sample cropped images from the proposed test dataset with eastern style headgears.

Table 8 presents the image and face-annotation distribution across the training, validation, and test splits of the proposed dataset. We split the local dataset to transfer learn the features from WIDER FACE dataset. Additionally, this local dataset demonstrates a wide range of face distances from the camera that affect the detector quality for detecting faces.

**Table 8:** Image and face-annotation distribution of the proposed dataset.

Split	Images	Positive images	Annotated faces	Negative images
Train	194	65	178	129
Validation	64	36	70	28
Test	178	64	102	114
**Total**	**436**	**165**	**350**	**271**

This dataset will play an important role in validating detector performance under local conditions involving different types of headgear.

## Results

We evaluated the proposed method and state-of-the-art methods on three datasets: WIDER FACE, VisDrone-DET2019, and our proposed local face dataset recorded under uncontrolled conditions.

### A. Evaluation metrics

The evaluation of the proposed detector is conducted using the mean average precision at a 50 percent intersection-over-union threshold (mAP@50) as the primary evaluation metric. In this metric, detections are matched with ground-truth annotations when the intersection-over-union (IoU) between the predicted and ground-truth bounding boxes is greater than or equal to 0.5.

The mean average precision at an IoU threshold of 0.5 (mAP@50) is determined by first calculating the average precision at IoU 0.5 for each individual class and then computing the mean across all classes. If *N* represents the total number of classes, mAP@50 is computed as shown in Equation 4.


$$\begin{array}{c} {mAP}_{50}=\frac{1}{N}\sum_{i=1}^{N}{AP}_{i,50} \end{array}$$ (4)

Equation 4 defines mAP@50 as the average of the class-wise average precision values computed at an IoU threshold of 0.5.

Here, N is the total number of classes, and the summation aggregates AP values from class 1 to N. The confidence threshold is varied from the lowest to the highest value in the confidence score vector. For each change in the confidence score, true positives, false positives, and false negatives are calculated. To compute mAP@50, the confidence threshold is varied incrementally from its minimum to its maximum value within the confidence score vector. At each threshold level, true positives (TP), false positives (FP), and false negatives (FN) are calculated. These values are then used to compute precision (PR) and recall (RC), using the following equations.


$$\begin{array}{c} PR=\frac{TP}{TP+FP} \end{array}$$ (5)

$$\begin{array}{c} RC=\frac{TP}{TP+FN} \end{array}$$ (6)

Equation 5 defines precision (PR) as the ratio of correctly predicted positive instances (TP) to all predicted positive instances (TP + FP), indicating the precision of positive predictions. Equation 6 defines recall (RC) as the ratio of correctly predicted positive instances (TP) to all actual positive instances (TP + FN), reflecting the model’s ability to identify all relevant instances.

For every variation in the confidence score threshold, a corresponding precision and recall pair is obtained. These pairs are plotted to form the precision–recall curve, and the area under the curve (AUC) is then computed. If k denotes the number of points on the PR curve, the AUC is determined using Equation 7.


$$\begin{array}{c} AUC=\sum_{i=1}^{k-1}\left({RC}_{i+1}-{RC}_{i}\right)\frac{{PR}_{i+1}+{PR}_{i}}{2} \end{array}$$ (7)

Equation 7 calculates the area under the precision–recall (PR) curve using the trapezoidal rule, which approximates the area under a curve represented by discrete points. Each point on the PR curve corresponds to a precision–recall pair at a particular threshold. The formula takes consecutive recall values, ${RC}_{i}$ and ${RC}_{i+1}$, and computes the area of the trapezoid between them. The base of the trapezoid is ${RC}_{i+1}-{RC}_{i}$, and its height is the average of the corresponding precision values, $({PR}_{i}+{PR}_{i+1})/2$. Summing these areas for *i* = 1 to k−1 estimates the total area under the PR curve. This area provides a single-number summary of classifier performance and is particularly useful for imbalanced datasets, where precision and recall are more informative than mAP@50 or ROC AUC.

### B. Results on the WIDER FACE dataset

The WIDER FACE dataset [2] is one of the well known datasets for face detection. It provides three settings for the evaluation of a face detector, namely Easy, Medium, and Hard. These evaluation settings help researchers to robustly evaluate the detector and know its resilience in different scenarios such as the size of the face, occlusions, and pose.

The easy evaluation setting consists of faces that are large relative to the image size, have minimal or no occlusion, and are mostly frontal. The task of the face detector usually becomes very simple in easy settings. In the medium evaluation setting, face sizes are in a normal range, i.e., not too small and not too big, and easily verifiable with the human eye. The occlusions are moderate and poses are also not challenging. mAP@50 achieved by the face detectors in the medium evaluation setting shows the resilience of the face detector under mildly challenging environments. Finally, in the hard evaluation setting, face sizes are very small as compared to the image sizes. In addition, the faces are also moderately to highly occluded along with challenging pose positions. This is the most difficult evaluation setting for the face detection model.

Our proposed method in all three categories of the WIDER FACE dataset scored higher than 80% mAP@50 along with other deep models for face detection, as shown in Fig 11. In the Easy and Medium settings, our proposed method achieves 93.07% mAP@50 and 91.25% mAP@50, respectively. In the Hard setting, our proposed method achieves 84.45% mAP@50.

**Fig 11:**
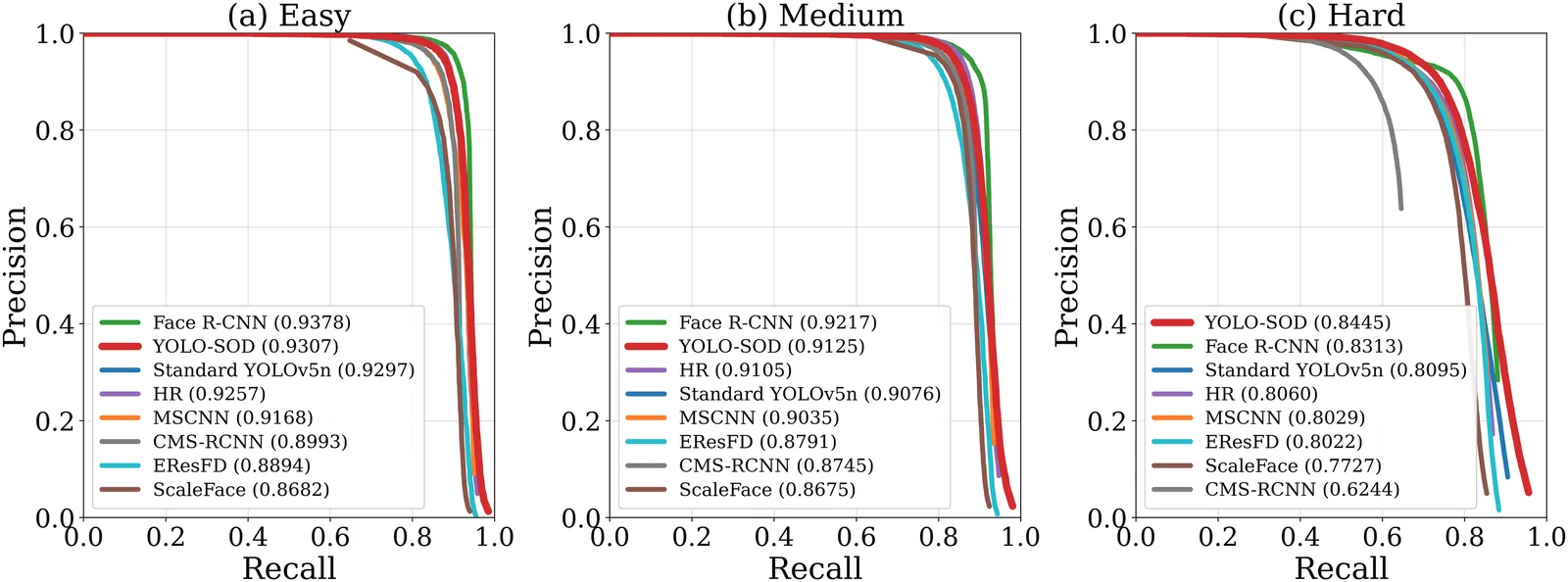
Precision–recall curves and mAP@50 results of YOLO-SOD on the WIDER FACE validation set: (a) Easy, (b) Medium, and (c) Hard subsets.

We observed that the mAP@50 of face detectors that were not robust to size variation and occlusion dropped significantly in the Hard evaluation setting, even though those detectors achieved above 90% mAP@50 in the Easy and Medium settings.

Table 9 compares YOLO-SOD with the standard YOLOv5 and YOLOv8 models on the Easy, Medium, and Hard subsets of the WIDER FACE dataset. It should be noted that the proposed method outperforms both YOLO variants, i.e., v5 and v8 in Medium and Hard subsets. However, the performance of YOLOv8 in Easy subset is better than that of the proposed method.

**Table 9:** Comparison of different methods on the WIDER FACE dataset at mAP@50.

Method	Easy	Medium	Hard
YOLOv5	0.929679	0.907638	0.809458
YOLOv8	**0.932543**	0.912321	0.818240
YOLO-SOD	0.930695	**0.912485**	**0.844502**

Table 10 shows the ablation study performed using the WIDER FACE dataset. YOLOv5 was considered as the baseline and then the progressive improvements show that the modified YOLOv5 architecture provides the main performance gain, while the Fuzzy Cr patches provide a smaller additional improvement over the mAP@50 and complement the whole small object detection pipeline very well.

**Table 10:** Ablation study on the WIDER FACE dataset at mAP@50.

Configuration	Easy	Medium	Hard
Baseline (YOLOv5)	0.929679	0.907638	0.809458
+ Modified YOLOv5 architecture	**0.930714**	0.912254	0.843574
+ Fuzzy Cr patches (YOLO-SOD)	0.930695	**0.912485**	**0.844502**

### C. Results on the proposed local test dataset

Single shot or YOLO-type detectors predict objects using grid-based feature maps. When several small or distant faces have centers within the same or neighboring grid locations, assignment and localization become more difficult. This limitation is especially relevant in dashcam scenes, where faces occupy only a very small proportion of the full image. The proposed dataset was intentionally not enriched with faces in every image because it would reduce its resemblance to real road conditions. Faces viewed from a vehicle are often sparsely distributed or visible only at distances ranging from a few to several tens of meters. Therefore, the proposed local dataset contains both sparse face and background only images. The background images strengthen the evaluation by testing whether the detector suppresses false positives when no face is present, while the annotated images evaluate its ability to detect small and distant faces.

The following results evaluate the proposed local dataset, which highlights local attire and variations in headgear styles. For evaluation, we divide all the faces into small, medium, and large classes, as shown in Table 11.

**Table 11:** Face scale groups for the proposed local dataset with an image resolution of 𝑤×h=1920×1080 pixels.

Face-scale group	Normalized face height	Bounding-box height
Small	h≤0.03	≤32.4 pixels
Medium	0.03<h≤0.06	> 32.4 to ≤64.8 pixels
Large	*h* > 0.06	> 64.8 pixels

As shown in Table 12, the overall mAP@50 values achieved by YOLOv5, YOLOv8, and YOLO-SOD are 45.63%, 46.51%, and 49.36%, respectively. In this paper, we used the Nano variants of all YOLO models with 2.5 million parameters. These models are GPU memory efficient and fast to train. Therefore, YOLOv5, YOLOv8, and YOLO-SOD represent the Nano version.

**Table 12:** Test mAP@50 on the proposed local dataset.

Method	mAP@50
YOLOv5	0.456311
YOLOv8	0.465078
**YOLO-SOD-ours**	**0.493615**

The mAP@50 may be further improved using models with more parameters, at the expense of GPU memory. Our proposed method achieves improvement of 3.73% over the YOLOv5 model and shows an improvement of 2.85% over the YOLOv8 model on the proposed local dataset.

We also ran the ablation studies on the local dataset and the results are shown in Table 13. The table shows that progressive improvements to the YOLOv5 architecture and detection technique enabled us to achieve 3.73% mAP@50 improvement over the standard YOLOv5. It also shows that the modified architecture provides the main improvement, while the other components provide smaller additional contributions to the overall mAP@50 performance.

**Table 13:** Ablation study of the proposed YOLO-SOD components on the local dataset.

Configuration	mAP@50
Baseline (YOLOv5)	0.456311
+ Modified YOLOv5 architecture	0.492982
**+ Fuzzy Cr patches (YOLO-SOD)**	**0.493615**

### D. Results on the VisDrone-DET2019 dataset

The VisDrone-DET2019 dataset [52] is one of the most popular datasets for the task of small object detection. It consists of ten object classes such as pedestrians, people, bicycle, car, van, truck, tricycle, awning-cycle, bus, and motor. The dataset has been recorded using a drone at varying altitudes. In addition, this dataset contains a wide variety of bounding box annotations (around 540,000 annotated bounding boxes) with different sizes. The dataset consists of 6471 training images, 548 validation images, and 1610 test images. Image sizes in the VisDrone-DET2019 dataset range from 960x540 pixels to higher resolutions.

These features enable researchers to test and evaluate the resilience of their detectors that are primarily designed for the purpose of small object detection. We also tested our proposed method on the VisDrone-DET2019 dataset. The motivation for testing our proposed method of detection on the VisDrone-DET2019 dataset is to evaluate the resilience of our detector for smaller objects. Faces also fall in the same size category of smaller objects relative to the image size. This has also been demonstrated in the sample and test figures of the proposed local test dataset. We turned off the color method for the detection of objects and the results are shown in the following Table 14.

**Table 14:** Evaluation of deep models on the VisDrone-DET2019 dataset.

Model	mAP@50 (test set)
YOLOv5 [53]	27.57
GBS YOLO [54]	35.3
HIC-YOLO [53]	36.95
SAHI [55]	43.5
YOLO-SOD-ours	46.76

During evaluation, we found that patching plays an important role in improving detector mAP@50 for small-object detection. SAHI [55] uses a similar technique known as slicing or tiling. Detectors that use this inference strategy can improve mAP@50 by approximately 5%–10% over state-of-the-art detector models. Our analysis suggests that this improvement results from the grid-based, anchor-based architecture of the detector. Before detection, predictions are produced from grid-based feature maps, with anchors assigned at each spatial location. When many small objects occupy a limited region, their centers may fall within the same or neighboring grid cells, making assignment and localization more difficult and resulting in missed detections.

Our proposed method [47] suggests dividing the image into patches before making the grid of blocks. This divides each image into smaller images, reducing the number of small objects represented within each local grid region. Our method gives 46.76% mAP@50 on the VisDrone-DET2019 dataset, which further bolsters the validity of our proposed method for the purpose of small object detection.

Furthermore, the proposed modifications in the backbone, neck, and head of the deep network for the detection of smaller objects relative to the image size also increased mAP@50. Fig 12 illustrates the qualitative performance of the proposed detection framework on the proposed local dataset.

**Fig 12:**
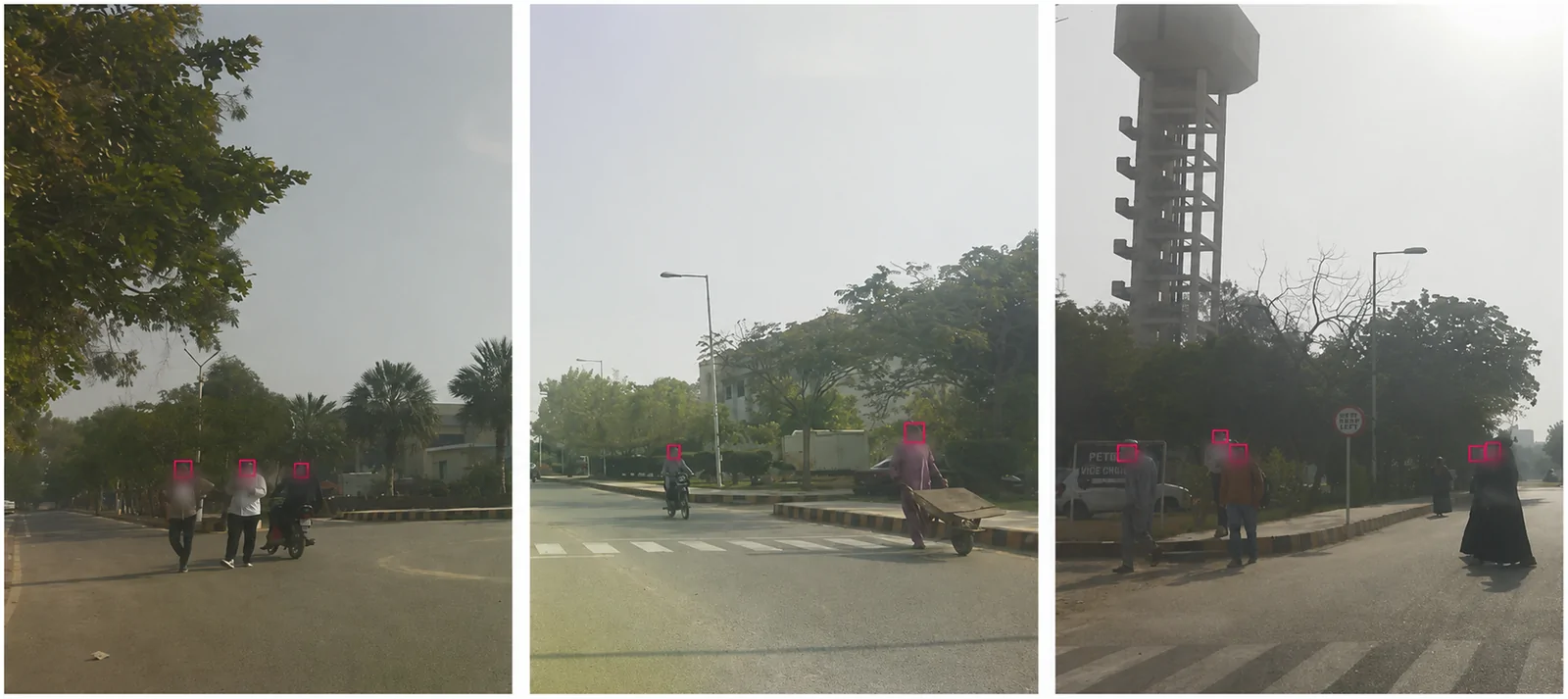
Performance of YOLO-SOD on the proposed local test dataset.

## Limitations

In this section, we discuss the possible limitations of our proposed method. The effectiveness of the Cr based color probability map may vary under different illumination conditions and skin color characteristics. Secondly, background regions with similar Cr responses may generate false clusters and affect patch selection strategy. In the case of extremely distant faces, the visual information is often insufficient for reliable detection; therefore, those faces were not included in the evaluation for the proposed dataset.

## Conclusion

This work improves small face detection by (i) augmenting YOLOv5 with additional convolutional blocks and specialized detection heads that leverage larger backbone feature maps, and (ii) introducing a dynamic patch size selection mechanism guided by Cr channel adaptive color probability maps and a lightweight Fuzzy inference system. The architectural changes increase the detector’s responsiveness or sensitivity to tiny targets, while the adaptive patching aligns the processing scale with scene content, benefiting both near and far faces.

Empirical evaluations confirm the effectiveness of the proposed components. The method achieves strong performance on WIDER FACE (93.07%, 91.25%, and 84.45% mAP@50 on the Easy, Medium, and Hard subsets, respectively), solid results on our dataset (49.36% mAP@50 for the proposed local dataset test set that includes faces of different sizes), and demonstrates effective generalization to small object detection on VisDrone-DET2019 (46.76 mAP@50). Together, these findings indicate that integrating high resolution feature pathways with color driven, fuzzy scale selection is a practical and accurate solution for small object scenarios.
