# Supplementary material for: YOLO-SOD: Color probability map-based fuzzy framework using deep models for face detection
Source: PLoS One. 2026 Oct 22;21(10):e0360794. doi: 10.1371/journal.pone.0360794 (PMC13690218; doi:10.1371/journal.pone.0360794)
Supplement: Supplementary file 1 [file pone.0360794.s001.pdf]

This study presents a noteworthy methodology for detecting small faces in images by integrating deep learning and fuzzy logic. Nevertheless, several concerns require attention before the work can be fully evaluated

Comments

- In the Abstract and Introduction section, the manuscript does not clearly establish its novelty. While multiple contributions are listed, it remains unclear what is fundamentally new compared to existing works. The paper lacks a strong positioning of its contribution as a unified system-level innovation, which weakens its impact.
- In the Methodology section, specifically the Fuzzy Inference System, the justification for using fuzzy logic is insufficient. The manuscript introduces rule-based decisions but does not explain why fuzzy logic is preferred over simpler thresholding or modern learning-based approaches, nor does it provide a comparative analysis.
- In the Color Probability Maps section, the assumption that the Cr channel effectively represents human skin is not rigorously justified. This approach may not generalize well across varying lighting conditions, skin tones, or environmental scenarios, and the manuscript lacks discussion of these limitations.
- In the Results section, the comparison with state-of-the-art methods is limited. The manuscript does not include recent models such as newer YOLO variants or transformer-based detectors, and it is unclear whether the comparisons are conducted under fair and consistent experimental settings.
- In the Custom Dataset description, the dataset design is not sufficiently justified. A large portion of images lack face annotations, which may weaken the reliability of the evaluation. The manuscript does not adequately explain how this affects performance assessment.
- Across the Methodology and Results sections, the manuscript lacks an ablation study. There is no clear breakdown of how each component (modified architecture, patching strategy, fuzzy system) contributes individually to the final performance, making it difficult to assess the effectiveness of each module.

- In the Introduction section, the writing style includes informal phrases such as 'in our opinion,' which are not suitable for academic writing. A more formal tone should be adopted throughout the manuscript.
- Throughout the manuscript, there are multiple grammatical and sentence structure inconsistencies. These issues affect readability and clarity, and the paper would benefit from thorough proofreading or professional editing.
- In several figures, particularly Figure 11, the explanations are insufficient. The captions and in-text descriptions do not clearly guide the reader in interpreting the results, which reduces the effectiveness of the visual content.
- In the Evaluation Metrics section, the equations are not consistently formatted. Some equations lack proper numbering or alignment, which makes them harder to follow.
- In the References section, there are repeated references (Ref 12 & 15). The manuscript would benefit from including more recent and diverse references to better position the work within current research.

- In Table 1, the formatting and values appear inconsistent and unclear. The table should be revised to ensure the information is presented accurately and easily interpreted.
