# Supplementary material for: YOLO-SOD: Color probability map-based fuzzy framework using deep models for face detection
Source: PLoS One. 2026 Oct 22;21(10):e0360794. doi: 10.1371/journal.pone.0360794 (PMC13690218; doi:10.1371/journal.pone.0360794)
Supplement: Supplementary file 2 [file pone.0360794.s003.docx]

**Response to the Academic Editor and Reviewers**

**Manuscript ID: PONE-D-25-65594**

*YOLO-SOD: Color probability map-based fuzzy framework using deep models for face detection*

**Dear Academic Editor and Reviewers,**

We sincerely thank the Academic Editor and both reviewers for their careful assessment and constructive comments. We have extensively revised the manuscript in light of the feedback. The revision clarifies the system level novelty, expands quantitative validation of the Cr-channel cues, explains the choice of fuzzy inference, adds direct baseline comparisons, and component wise ablation studies, clarifies the local dataset design and split, standardizes the evaluation terminology and equations, and improves the figures, tables, references, and overall presentation. The detailed point-by-point responses are provided below. Page and line references correspond to the revised clean manuscript dated August 09, 2026.

**Evaluation clarification and regenerated graphs**

As part of the revision, we checked the evaluation pipeline and identified an implementation inconsistency in the earlier AP calculation. The affected values and plots were recomputed and the corresponding Wider Face and local dataset graphs, tables, Abstract, Results, and Conclusion were updated. For WIDER FACE, evaluation uses the official Easy/Medium/Hard .mat annotations and the standard 1,000-threshold AP protocol at IoU 0.50 through a Python implementation of the official MATLAB evaluator; comparison graphs load the official baseline .mat precision-recall curves. For the local dataset, a common VOC/YOLO-style AP@0.50 evaluator is applied identically to YOLOv5, YOLOv8, and YOLO-SOD using confidence ranked detections, one-to-one ground-truth matching, and precision-recall curve integration. This standardization ensures that all reported comparisons use consistent evaluation rules.

# A. Response to the Academic Editor and Journal Requirements

| **Comment** | **Response and location of revision** |
| --- | --- |
| **Journal requirement 1** *Ensure that the manuscript meets PLOS ONE style and file-naming requirements.* | The manuscript has been reformatted using the current PLOS ONE LaTeX template.  **Location in revised manuscript:** Formatting changes apply throughout the manuscript. |
| **Journal requirement 2** *Author-generated code underpinning the findings must be shared without restriction and in a reproducible form.* | The code has been uploaded and shared at GITHUB link https://github.com/YawarGuguma/color-probability-maps |
| **Journal requirements 3–4** *Clarify restrictions on data sharing or deposit the minimal anonymized dataset in a stable public repository.* | Dataset has been uploaded here  https://doi.org/10.6084/m9.figshare.33189120  Password is 404 |
| **Journal requirement 5** *Funding information and the financial disclosure do not match; provide the correct grant information.* | This is the PhD study and supported through NED research fund. The acknowledgement has been included inside the paper. |
| **Journal requirement 6** *State the role of the funder.* | Funder: NED University of Engineering and Technology (NEDUET)  NEDUET supported this study because the scholar is a PhD student at NEDUET and striving to complete her PhD. |
| **Journal requirement 7** *Figures 1, 2, 3, 4, 5, 6, 7, 8, 10, and 13 were flagged for possible copyrighted material.* | Emails were sent to take permission but did not received the reply.  Therefore, the copyrighted material has been removed please. The datasets are public and the reader may download and see the dataset images |
| **Journal requirement 8** *Refer to Figures 9, 11, and 12 in the manuscript text.* | Authors want to thank the editor for this comment. All Figures have been cited please. |
| **Journal requirement 9** *Figures containing identifiable participants require specific written consent for publication under the PLOS CC BY license.* | We acknowledge the journal’s participant consent requirement. Identifiable participant images will be retained only where the required PLOS publication consent has been obtained and securely filed. The ethics/methods statement will explicitly state that written informed consent for publication under the PLOS terms was obtained. Where consent is unavailable, the relevant image will be removed or replaced with a non-identifying visualization or blurring. |
| **Journal requirement 10** *Refer to Tables 1 and 2 in the manuscript text.* | Both tables are now cited in the body text. Thank you. |
| **Journal requirement 11** *Evaluate reviewer-recommended literature and cite it only when relevant.* | Acknowledged and noted. No references were suggested by the reviewers. Thank you. |
| **Additional Editor Comment** *Please revise as per comments.* | The manuscript has been comprehensively revised in response to all journal requirements and reviewer comments. The specific revisions are itemized below. |

# B. Response to Reviewer 1 (p is page; pp is pages)

| **Comment** | **Response and location of revision** |
| --- | --- |
| **Reviewer 1, Comment 1** *The novelty appears incremental because the method combines known YOLO modifications, tiling, color preprocessing, and fuzzy inference. Clarify the unique contribution.* | We agree that the novelty needed clearer positioning. The revised manuscript now presents the contribution as a unified system level solution rather than claiming that each component is individually. The distinctive contribution is the joint use of (i) higher resolution YOLOv5 feature pathways and small object detection heads and (ii) image-adaptive patch configuration inferred from Cr cluster count and scale cues through a lightweight FIS. Unlike fixed tiling, the number and size of patches are selected separately for each image. Component wise ablations were also added to quantify the contribution of the architecture and adaptive fuzzy patching.  **Location in revised manuscript:** Abstract, p. 1; Introduction, pp. 2–3, lines 27–65; Problem identification, p. 5, lines 174–192; WIDER ablation, Table 7; local ablation, Table 10. |
| **Reviewer 1, Comment 2** *The Cr-channel assumptions are insufficiently validated: clusters may not correspond to faces, density may not correlate with distance, and representation may vary across lighting, ethnicity, and cameras.* | We substantially expanded this analysis. First, a head region contrast score was computed across RGB, HSV, and YCbCr channels using 500 aligned WIDER FACE crops; Cr achieved the highest Head region Contrast Score (1.818). Second, an image level analysis on 136 WIDER FACE images (1,310 faces) showed that images with more annotated faces generally produced more Cr candidate clusters, while explicitly stating that not every cluster is a face. Third, face scale groups were defined by normalized bounding box height, and Cr cluster size increased across small, medium, and large face groups. The manuscript now clearly states that cluster count and density are coarse patch selection cues, not direct estimates of face count or physical distance. A limitation statement also clarifies that invariance across skin tone, illumination, and camera characteristics is not established.  **Location in revised manuscript:** Cr channel analysis, pp. 11–15, lines 319–420; Fig. 5; Tables 1–3; limitation statement on p. 13, lines 366–368. |
| **Reviewer 1, Comment 3(a)** *The proposed dataset is small, non-public, and lacks sufficient statistical diversity.* | The revised manuscript now gives complete dataset statistics, positive and negative image counts, annotated face counts, and separate train/validation/test splits. The rationale for retaining background only images is also explained: they reflect realistic dashcam conditions and test false positive suppression when no face is present. The limitation associated with dataset scale is acknowledged through the more cautious interpretation of the local results.  **Location in revised manuscript:** Proposed dataset, pp. 19–20, lines 464–490; Tables 4 and 5; local test rationale, p. 24, lines 555–564. |
| **Reviewer 1, Comment 3(b)** *Comparisons with recent detectors, including newer YOLO variants, are missing.* | We added a controlled comparison with standard YOLOv5 and YOLOv8 on both WIDER FACE and the local proposed dataset.  **Location in revised manuscript:** Table 6, pp. 23–24; local comparison, Table 9, p. 24. |
| **Reviewer 1, Comment 3(c)** *Real-time claims are unsupported by inference-speed or computational-cost analysis.* | We have avoided presenting YOLO-SOD as experimentally verified to be real-time. References to real-time behavior now describe previously published baseline architectures rather than a measured claim for the proposed method. The revised experimental discussion focuses on mAP50, uses Nano YOLO variants for a controlled comparison, and describes computational considerations qualitatively without reporting FPS claims.  **Location in revised manuscript: L**ocal results, p. 24, lines 568–575. |
| **Reviewer 1, Comment 4** *The Introduction is overly verbose and contains generic AI background.* | The Introduction was condensed and reorganized around the specific small face detection problem.  **Location in revised manuscript:** Introduction, pp. 2–3, lines 1–68. |
| **Reviewer 1, Comment 5** *The qualitative color maps and heatmaps should be supported by quantitative analysis.* | Quantitative validation was added. The revised manuscript reports channel wise Head region Contrast Score values, image level Cr cluster abundance statistics, face level cluster size statistics, and the proportion of annotated faces associated with at least one candidate cluster. These analyses directly support and bound the interpretation of Figures 5 and 6.  **Location in revised manuscript:** pp. 12–15, lines 352–420; Tables 1–3. |
| **Reviewer 1, Comment 6** *Terminology such as “accuracy” is used inconsistently instead of AP or mAP.* | The primary metric is now explicitly defined as mAP50. General uses of the word “accuracy” are intended only as broad meaning and not as a separate metric.  **Location in revised manuscript:** Evaluation Metrics, p. 22, lines 496–527; Tables 6–11; Fig. 11. |
| **Reviewer 1, Comment 7** *Dataset statistics in Table 1 are inconsistent and require clarification.* | The former dataset statistics presentation was corrected and expanded. The revised manuscript now separates overall dataset information from split-level statistics. Thank you  **Location in revised manuscript:** Tables 4 and 5, pp. 19–20, lines 471–490. |
| **Reviewer 1, Comment 8** *Grammar and stylistic issues require careful proofreading.* | The manuscript was extensively revised and edited for grammar and sentence structure.  **Location in revised manuscript:** Changes throughout the manuscript. |
| **Evaluation recalculation** *The evaluation protocol and result reliability require clarification.* | During the revision, an implementation inconsistency in the earlier mAP50 calculation was corrected and all affected results and plots were regenerated. WIDER FACE now uses official Easy/Medium/Hard .mat annotations and the standard 1,000-threshold protocol at IoU 0.50 through a Python implementation of the official MATLAB evaluator. The local dataset uses the same confidence ranked, one-to-one matching, PR-integration evaluator for YOLOv5, YOLOv8, and YOLO-SOD. The corrected WIDER FACE values are 93.07%, 91.25%, and 84.45% for Easy, Medium, and Hard; the corrected local YOLO-SOD value is 49.36%.  **Location in revised manuscript:** Abstract, p. 1; Evaluation Metrics, p. 22; WIDER FACE results, pp. 23–24, Fig. 11 and Tables 6–7; local results, pp. 24–25, Tables 9–10; Conclusion, p. 26. |

# C. Response to Reviewer 2

| **Comment** | **Response and location of revision** |
| --- | --- |
| **Reviewer 2, Comment 1** *The Abstract and Introduction do not clearly establish novelty or position the work as a unified system-level contribution.* | The Abstract and Introduction were rewritten to frame the work as a unified small-face detection framework. The revised positioning emphasizes the interaction between high resolution detection pathways from YOLO backbone, neck, and head to image adaptive fuzzy patch selection. The unique contribution is also stated explicitly in the Problem identification subsection and supported by ablation studies.  **Location in revised manuscript:** Abstract, p. 1; Introduction, pp. 2–3, lines 27–65; Problem identification, p. 5, lines 174–192; Tables 7 and 10. |
| **Reviewer 2, Comment 2** *The manuscript does not justify why fuzzy logic is preferred over hard thresholds or a learned selection model.* | A dedicated justification was added. The revised text explains that fixed threshold ranges require extensive manual tuning, whereas an additional learned model would introduce extra complexity and supervision solely for patch selection. The Fuzzy system provides a lightweight way to combine cluster count and density through overlapping membership functions and interpretable rules.  **Location in revised manuscript:** Fuzzy patches subsection, p. 16, lines 421–429, followed by the five fuzzy rules at lines 439–444. |
| **Reviewer 2, Comment 3** *The Cr channel may not generalize across lighting, skin tones, or environmental conditions, and the limitations are not discussed.* | The revised manuscript adds quantitative support while explicitly limiting the claim. Cr is treated only as a heuristic scene level cue for patch selection, not as a skin classifier or face detector. The text states that WIDER FACE lacks explicit metadata for skin tone, illumination, and camera characteristics, and therefore invariance across these factors is not established.  **Location in revised manuscript:** Cr channel section, pp. 11–15, especially p. 13, lines 366–368, and pp. 14–15, lines 372–420. |
| **Reviewer 2, Comment 4** *The Results comparison is limited and the fairness of comparisons is unclear.* | We added comparisons with YOLOv5 and YOLOv8, using consistent model scale and evaluation procedures. WIDER FACE results are reported on the official Easy, Medium, and Hard validation subsets. Figure 11 uses official baseline .mat precision-recall curves for literature methods, while Tables 6 and 9 distinguish the directly evaluated baselines.  **Location in revised manuscript:** WIDER results, pp. 23–24; local results, p. 24. |
| **Reviewer 2, Comment 5** *The custom dataset design is insufficiently justified, particularly the large number of images without faces.* | The revised manuscript explains that background-only images were intentionally retained to reproduce real dashcam conditions, where faces are sparse or absent. These images test whether the detector suppresses false positives, while positive images evaluate small and distant face detection. The complete positive/negative distribution and split statistics are now reported.  **Location in revised manuscript:** Dataset description, pp. 19–20, lines 471–490; local-results rationale, p. 24, lines 555–564; Tables 4 and 5. |
| **Reviewer 2, Comment 6** *The manuscript lacks an ablation study for the modified architecture, patching strategy, and fuzzy system.* | Two component wise ablation studies were added. Table 7 reports the WIDER FACE progression from baseline YOLOv5 to the modified architecture and then to fuzzy Cr patches. Table 10 reports the same progression on the local dataset.  **Location in revised manuscript:** Table 7, p. 24, lines 552–554; Table 10, p. 25, lines 576–579. |
| **Reviewer 2, Comment 7** *Informal phrases such as “in our opinion” should be replaced by formal academic wording.* | The manuscript has been substantially revised toward a more formal tone |
| **Reviewer 2, Comment 8** *Grammatical and sentence-structure inconsistencies affect readability.* | The manuscript was extensively proofread and edited for grammar, sentence structure, and clarity.  **Location in revised manuscript:** Changes throughout the manuscript. |
| **Reviewer 2, Comment 9** *Figure 11 and several other figures require clearer captions and in-text explanations.* | Figure 11 was replaced with three clearly labeled precision-recall panels for the Easy, Medium, and Hard subsets. All other figures were reviewed and their caption were modified where necessary  **Location in revised manuscript:** Figure 11 and WIDER Results, p. 23, lines 541–547; Figures 5–6 and supporting Tables 1–3, pp. 12–15. |
| **Reviewer 2, Comment 10** *Equations in the Evaluation Metrics section are inconsistently formatted or numbered.* | The evaluation equations were reformatted as numbered display equations and explained immediately after presentation. The revised section defines mAP50, precision, recall, and PR-curve integration in a consistent sequence.  **Location in revised manuscript:** Evaluation Metrics, p. 22, lines 496–527; Equations 4–7. |
| **Reviewer 2, Comment 11** *The References contain a duplicate and should include more recent and diverse work.* | The duplicate reference was removed and the bibliography was checked for unique entries.  **Location in revised manuscript:** Related Work, pp. 3–5; References, pp. 26–30. |
| **Reviewer 2, Comment 12** *Table 1 formatting and values are inconsistent and unclear.* | The tables were reorganized and clarified. The revised Table 1 now reports a clearly defined quantitative Head region Contrast Score analysis.  The former dataset statistics were corrected and expanded into Tables 4 and 5 so that totals and split-level values are transparent and internally consistent.  **Location in revised manuscript:** Table 1, p. 13; Tables 4–5, pp. 19–20. |
| **Corrected WIDER/local graphs** *The revised result plots should be based on a consistent and clearly stated evaluation procedure.* | The WIDER FACE and local result graphs were regenerated after standardizing the metric implementation. WIDER FACE uses official subset annotations and the standard 1,000-threshold evaluator at IoU 0.50, and the local comparison uses one identical evaluator for all three methods. The revised values are consistently propagated to the Abstract, result tables, Figure 11, and Conclusion.  **Location in revised manuscript:** Abstract, p. 1; Evaluation Metrics, p. 22; WIDER Results, pp. 23–24; local Results, pp. 24–25; Conclusion, p. 26. |

**Sincerely,**

**Ms. Aniqa Saba,**

**Prof. Dr. Muhammad Faizan Shirazi,**

**Prof. Dr. Yawar Rehman,**

**Prof. Dr. Muhammad Mubashir Khan**
