# Supplementary material for: YOLO-SOD: Color probability map-based fuzzy framework using deep models for face detection
Source: PLoS One. 2026 Oct 22;21(10):e0360794. doi: 10.1371/journal.pone.0360794 (PMC13690218; doi:10.1371/journal.pone.0360794)
Supplement: Supplementary file 3 [file pone.0360794.s004.docx]

**Response to the Academic Editor and Reviewers**

**Manuscript ID: PONE-D-25-65594R1**

**YOLO-SOD: Color probability map-based fuzzy framework using deep models for face detection**

Dear Academic Editor and Reviewers,

We thank you for your valuable comment for improving our paper. Following we present point by point response and the exact location of each revision.

**Additional comments to Editor**

It is to inform that the affiliation of author Yawar Rehman’s has been updated. Thank you.

# **A. Response to the Academic Editor and Journal Requirements**

| **Comment** | **Response and location of revision** |
| --- | --- |
| **Academic Editor Comment** *Please critically resolve these minor issues.* | Authors thank the editor and all the reviewers for their efforts for the improvement of our paper. We have critically analyzed and resolved the highlighted minor issues. |
| **Journal requirement - References** *Please review your reference list to ensure that it is complete and correct. If you have cited papers that have been retracted, please include the rationale for doing so in the manuscript text, or remove these references and replace them with relevant current references. Any changes to the reference list should be mentioned in the rebuttal letter that accompanies your revised manuscript. If you need to cite a retracted article, indicate the article's retracted status in the References list and also include a citation and full reference for the retraction notice.* | Thank you for this comment. We carefully reviewed the complete reference list. None of the reference is found to be retracted. |

# **B. Response to Reviewer 1**

| **Comment** | **Response and location of revision** |
| --- | --- |
| **Reviewer 1, Comment 1** *Correct the WIDER FACE result inconsistency. The Abstract reports 83.4%, 84.8%, and 70.4% for Easy/Medium/Hard, while the Results section reports 83.4%, 84.4%, and 70.4%. Choose the verified value and use it consistently throughout the Abstract, Results, tables, and Conclusion.* | We think there has been some confusion because the results reported in the submitted revision 1 for Wider Face dataset are 93.07% mAP50 (Easy), 91.25% mAP50 (Medium), and 84.45% mAP50 (Hard) are consistent throughout the manuscript. |
| **Reviewer 1, Comment 2** *Correct Table 1. There appears to be a serious formatting/numerical corruption in the dataset table. The text says there are 436 images and 350 face annotations, but Table 1 appears as "35024," "545," and "88," which is clearly inconsistent with the surrounding description. Verify and reconstruct the table from the original dataset records before resubmission.* | We think there has been some confusion because Table 1 in the submitted revision 1 represents contrast score analysis for different datasets.  And the values that the reviewer mentioned in this comment pertains to Table 4 for submitted revision 1, which is accurate with its surrounding explanation. |
| **Reviewer 1, Comment 3** *Clarify the annotation counts. The manuscript states: - 436 total images - 271 images with no faces - 83 images with one face - 67 images with 2-4 faces - 12 images with 5-7 faces - 3 images with 8-10 faces These image counts sum to 436, which is good, but the stated 350 annotations should be reconciled mathematically with these categories. Add a short explanation of exactly how the 350 annotations were calculated.* | Table 8: Distribution of images and face annotations in the proposed dataset, has been included to reconcile the 436 image with 350 annotations. |
| **Reviewer 1, Comment 4** *Use consistent terminology throughout. Standardize: - YOLOv5 rather than alternating between Yolo, Yolo5, YOLO5, and YOLO v5 - WIDER FACE rather than Wider Face/Wider dataset - VisDrone-DET2019 consistently - mAP@50 or AP50 consistently - "color probability map" consistently - "patch size" rather than occasionally "path size" or "patches size."* | We thank you reviewer for this comment. All the terminologies in the manuscript has been made consistent as suggest by the reviewer. |
| **Reviewer 1, Comment 5** *Correct the title terminology. The title currently says: "YOLOv5-Small Object Detection: Color Probability Map Based Fuzzy Framework Using Deep Models for Face Detection" A cleaner version would be: "YOLO-SOD: A Color-Probability-Map-Based Fuzzy Framework for Small-Face Detection Using YOLOv5" This better reflects the actual contribution.* | We thank reviewer for this comment. However, this paper is related to the approved PhD title from ASRB board of NED university, changing the title of the paper would complicate things for the PhD degree please. We sincerely hope and request understanding on this matter. Thank you.  Our current title of the paper is as follows as submitted in the revision 1.  “YOLO-SOD: Color probability map-based fuzzy framework using deep models for face detection” |
| **Reviewer 1, Comment 6** *Improve the description of the YOLOv5 architecture. The manuscript says the default YOLOv5 has three heads and later says the proposed model has four heads, while also describing removal/addition of heads. This should be presented in a concise table: Component \| Original YOLOv5 \| Proposed YOLO-SOD Detection heads \| 3 \| 4 Feature maps \| 80x80, 40x40, 20x20 \| 320x320, 160x160, 80x80, 40x40 Added C3 blocks \| - \| 4 20x20 head \| Present \| Removed* | Table 1: Comparison of the original YOLOv5 and the proposed YOLO-SOD architecture, has been added to comply this comment. |
| **Reviewer 1, Comment 7** *The current architectural description supports this information but makes the reader reconstruct it themselves.* | This comment has been addressed in the response of Comment 6 of the reviewer. |
| **Reviewer 1, Comment 8** *Explain the fuzzy membership functions quantitatively. The five fuzzy rules are given, but the manuscript does not adequately specify the membership functions/ranges for "low," "medium," and "high" cluster number/density. Add a small table specifying: - input variables, - membership ranges, - membership-function type, - output patch sizes, - defuzzification method.* | Table 5: Quantitative specification of the fuzzy inference system used for adaptive patch selection, has been added to address the reviewer’s comment. |
| **Reviewer 1, Comment 9** *Tone down unsupported statements. For example: "current state-of-the-art deep models ... may fail to detect the faces in the wild" and "conventional state-of-the-art networks often fail." These are stronger than necessary. Replace with evidence-based wording such as: "Performance may decrease under unconstrained conditions involving small face size, occlusion, pose variation, and complex backgrounds." The manuscript itself identifies these conditions as challenges.* | The highlighted statements have been toned down as suggested by the reviewer. |
| **Reviewer 1, Comment 10** *Replace subjective wording. Phrases such as: - "In our opinion" - "One sentence answer is..." - "very good clues" - "golden 80% accuracy mark" - "intelligent, cluster-aware modification" - "promising transfer" should be changed to objective scientific language. For example, "golden 80% accuracy mark" should simply be removed.* | The subjective wordings has been replaced with scientific language as suggested by the reviewer. |
| **Reviewer 1, Comment 11** *Strengthen the limitations section. The manuscript acknowledges that far-distance faces were excluded because of poor image quality. This should explicitly appear in the Limitations section, together with: - dependence of the Cr-based map on illumination/skin-color characteristics, - possible false clusters caused by background regions, - limited size of the proposed local dataset, - lack of evaluation for extremely distant faces.* | Limitations Section has been added as suggested by the reviewer. |
| **Reviewer 1, Comment 12** *Clarify the local dataset methodology. Include, if available: - where/how images were collected, - collection period, - camera/device information, - selection criteria, - annotation procedure, - annotator verification, - whether the dataset is publicly available. This is particularly important because the local dataset is presented as a contribution.* | Statements about the data collection has been added in Section E of Proposed Methodology titled as ‘Proposed dataset highlighting local attire conditions’ as suggested by the reviewer. |
| **Reviewer 1, Comment 13** *Improve the evaluation description. The manuscript explains mAP/AP at considerable length, but some of the discussion is unnecessarily textbook-like. For a research article, this can be shortened and replaced with the exact evaluation protocol used in the experiments.* | We thank the reviewer for this valuable suggestion. After careful consideration, we believe that implementing the suggested change may reduce the clarity and interpretability of the manuscript. Therefore, we have retained the current presentation and respectfully hope that the reviewer will understand our rationale. |
| **Reviewer 1, Comment 14** *Add an ablation study if the experimental data are available. This would substantially strengthen the paper without changing the methodology. At minimum: - baseline YOLOv5 - YOLOv5 + modified architecture - YOLOv5 + fixed patching - YOLOv5 + fuzzy patch selection - complete YOLO-SOD This would demonstrate whether the improvement comes from the architectural modification, the patching, the fuzzy system, or their combination.* | We thank the reviewer for this valuable suggestion, which would further strengthen the quality of the work. We plan to conduct this ablation study in our future extension of this research, where the proposed method will be evaluated through hardware implementation. |
| **Reviewer 1, Comment 15** *Correct grammar throughout. There are many recurring grammatical problems, for example: "These feature maps works perfect..." -> "These feature maps work well..." "the detection head ... get the feature map" -> "the detection head ... receives the feature map" "We observed that the accuracy..." -> "We observed that detector accuracy..." "faces at near to medium distance" -> "faces at near-to-medium distances" "patches plays an important" -> "patches play an important role"* | Manuscript has been carefully proofread and grammar has been corrected as suggested by the reviewer. |
| **Reviewer 1, Comment 16** *Correct the Conclusion formatting. The Conclusion currently contains an apparent stray word: "Use This work improves..." This should be removed. Also insert spaces between the reported values: "83.4%84.8%70.4 AP" should be: "83.4%, 84.8%, and 70.4% AP".* | We think there has been some confusion. We checked thoroughly and found that our submitted manuscript as revision 1 does not contains these errors. |
| **Reviewer 1, Highest-priority fixes** *If you want this to remain a minor revision, I would prioritize these five: 1. Fix Table 1 and reconcile the dataset numbers. 2. Resolve 84.8% vs 84.4% for WIDER FACE. 3. Standardize YOLOv5/WIDER FACE/VisDrone/mAP terminology. 4. Add the fuzzy membership-function details. 5. Perform a thorough English/grammar and scientific-style edit. The underlying contribution - modified YOLOv5 architecture + adaptive patching + Cr-based clustering + FIS - is already clearly described in the manuscript. I would not make major changes to the proposed method unless the reviewer specifically requests additional experiments.* | We check and rectified all the identified issues as suggest by the reviewer. |

# **C. Response to Reviewer 2**

| **Comment** | **Response and location of revision** |
| --- | --- |
| **Reviewer 2, Comment 1** *Please use AP/mAP terminology consistently throughout the manuscript. In particular, Table 9 is titled "Test accuracy" although the reported metric is mAP50, and there are still sentences that describe mAP50 values as "accuracy." These should be revised simply to report the results as mAP50.* | We thank the reviewer for this comment. We have correctly reported the results as mAP@50 instead of writing accuracy as suggested by the reviewer. |
| **Reviewer 2, Comment 2** *The interpretation of the ablation results should be slightly moderated. The modified YOLOv5 architecture provides the dominant improvement, while the additional fuzzy Cr-based patching stage gives a smaller incremental gain. Therefore, statements such as "each module improves the accuracy" should be rephrased so that they accurately reflect the values in Tables 7 and 10. I do not consider additional experiments essential here; a more precise discussion of the reported results would be sufficient.* | We thank the reviewer for this comment. We have moderated the discussions on the mentioned places of the reviewer. |
| **Reviewer 2, Comment 3** *A final careful language edit is still recommended. There are several remaining grammatical and typographical issues (for example, expressions such as "fastly trainable," "the background strengthen," and similar wording). Cleaning these up would improve the readability and presentation of the final manuscript.* | We thank the reviewer for this comment. We have carefully corrected and cleaned up the grammatical and typographical errors as suggested by the reviewer. |

Sincerely,

Ms. Aniqa Saba,

Prof. Dr. Muhammad Faizan Shirazi,

Prof. Dr. Yawar Rehman,

Prof. Dr. Muhammad Mubashir Khan
