# Supplementary material for: YOLO-SOD: Color probability map-based fuzzy framework using deep models for face detection
Source: PLoS One. 2026 Oct 22;21(10):e0360794. doi: 10.1371/journal.pone.0360794 (PMC13690218; doi:10.1371/journal.pone.0360794)
Supplement: Peer review history

# Peer review: Decision Letter 0

**DOI:** 10.1371/journal.pone.0360794.r001

**Authors:** Usman Ghafoor

3 May 2026

Dear Dr. Shirazi,

Thank you for submitting your manuscript to PLOS ONE. After careful consideration, we feel that it has merit but does not fully meet PLOS ONE’s publication criteria as it currently stands. Therefore, we invite you to submit a revised version of the manuscript that addresses the points raised during the review process.

Please submit your revised manuscript by Jun 17 2026 11:59PM. If you will need more time than this to complete your revisions, please reply to this message or contact the journal office at plosone@plos.org. When you're ready to submit your revision, log on to https://www.editorialmanager.com/pone/ and select the 'Submissions Needing Revision' folder to locate your manuscript file.

- A letter that responds to each point raised by the academic editor and reviewer(s). You should upload this letter as a separate file labeled 'Response to Reviewers'.
- A marked-up copy of your manuscript that highlights changes made to the original version. You should upload this as a separate file labeled 'Revised Manuscript with Track Changes'.
- An unmarked version of your revised paper without tracked changes. You should upload this as a separate file labeled 'Manuscript'.

If you would like to make changes to your financial disclosure, please include your updated statement in your cover letter. Guidelines for resubmitting your figure files are available below the reviewer comments at the end of this letter.

If applicable, we recommend that you deposit your laboratory protocols in protocols.io to enhance the reproducibility of your results. Protocols.io assigns your protocol its own identifier (DOI) so that it can be cited independently in the future. For instructions see: https://journals.plos.org/plosone/s/submission-guidelines#loc-laboratory-protocols. Additionally, PLOS ONE offers an option for publishing peer-reviewed Lab Protocol articles, which describe protocols hosted on protocols.io. Read more information on sharing protocols at https://plos.org/protocols?utm_medium=editorial-email&utm_source=authorletters&utm_campaign=protocols.

As the corresponding author, your ORCID iD is verified in the submission system and will appear in the published article. PLOS supports the use of ORCID, and we encourage all coauthors to register for an ORCID iD and use it as well. Please encourage your coauthors to verify their ORCID iD within the submission system before final acceptance, as unverified ORCID iDs will not appear in the published article. *Only*  the individual author can complete the verification step; PLOS staff *cannot*  verify ORCID iDs on behalf of authors.

We look forward to receiving your revised manuscript.

Kind regards,

Usman Ghafoor

Academic Editor

PLOS One

Journal Requirements:

When submitting your revision, we need you to address these additional requirements.

1. Please ensure that your manuscript meets PLOS ONE's style requirements, including those for file naming. The PLOS ONE style templates can be found at

https://journals.plos.org/plosone/s/file?id=wjVg/PLOSOne_formatting_sample_main_body.pdf and https://journals.plos.org/plosone/s/file?id=ba62/PLOSOne_formatting_sample_title_authors_affiliations.pdf

2. Please note that PLOS One has specific guidelines on code sharing for submissions in which author-generated code underpins the findings in the manuscript. In these cases, we expect all author-generated code to be made available without restrictions upon publication of the work. Please review our guidelines at https://journals.plos.org/plosone/s/materials-and-software-sharing#loc-sharing-code and ensure that your code is shared in a way that follows best practice and facilitates reproducibility and reuse.

3. We note that you have indicated that there are restrictions to data sharing for this study. PLOS only allows data to be available upon request if there are legal or ethical restrictions on sharing data publicly. For more information on unacceptable data access restrictions, please see http://journals.plos.org/plosone/s/data-availability#loc-unacceptable-data-access-restrictions.

Before we proceed with your manuscript, please address the following prompts:

a) If there are ethical or legal restrictions on sharing a de-identified data set, please explain them in detail (e.g., data contain potentially identifying or sensitive patient information, data are owned by a third-party organization, etc.) and who has imposed them (e.g., a Research Ethics Committee or Institutional Review Board, etc.). Please also provide contact information for a data access committee, ethics committee, or other institutional body to which data requests may be sent.

b) If there are no restrictions, please upload the minimal anonymized data set necessary to replicate your study findings to a stable, public repository and provide us with the relevant URLs, DOIs, or accession numbers. For a list of recommended repositories, please see

https://journals.plos.org/plosone/s/recommended-repositories. You also have the option of uploading the data as Supporting Information files, but we would recommend depositing data directly to a data repository if possible.

We will update your Data Availability statement on your behalf to reflect the information you provide.

4. In the online submission form, you indicated that data will be available upon request.

All PLOS journals now require all data underlying the findings described in their manuscript to be freely available to other researchers, either 1. In a public repository, 2. Within the manuscript itself, or 3. Uploaded as supplementary information.

This policy applies to all data except where public deposition would breach compliance with the protocol approved by your research ethics board. If your data cannot be made publicly available for ethical or legal reasons (e.g., public availability would compromise patient privacy), please explain your reasons on resubmission and your exemption request will be escalated for approval.

5. We note that the grant information you provided in the ‘Funding Information’ and ‘Financial Disclosure’ sections do not match.

When you resubmit, please ensure that you provide the correct grant numbers for the awards you received for your study in the ‘Funding Information’ section.

6. Thank you for stating the following financial disclosure:

This work was supported by research grant from NED University of Engineering & Technology, Karachi, Pakistan.

Please state what role the funders took in the study. If the funders had no role, please state: "The funders had no role in study design, data collection and analysis, decision to publish, or preparation of the manuscript."

If this statement is not correct you must amend it as needed.

Please include this amended Role of Funder statement in your cover letter; we will change the online submission form on your behalf.

7. We note that Figure(s) 1, 2, 3, 4, 5, 6, 7, 8, 10, 13 in your submission contain copyrighted images. All PLOS content is published under the Creative Commons Attribution License (CC BY 4.0), which means that the manuscript, images, and Supporting Information files will be freely available online, and any third party is permitted to access, download, copy, distribute, and use these materials in any way, even commercially, with proper attribution. For more information, see our copyright guidelines: http://journals.plos.org/plosone/s/licenses-and-copyright.

We require you to either (1) present written permission from the copyright holder to publish these figures specifically under the CC BY 4.0 license, or (2) remove the figures from your submission:

a. You may seek permission from the original copyright holder of Figure(s) 1, 2, 3, 4, 5, 6, 7, 8, 10, 13 to publish the content specifically under the CC BY 4.0 license.

We recommend that you contact the original copyright holder with the Content Permission Form (http://journals.plos.org/plosone/s/file?id=7c09/content-permission-form.pdf) and the following text:

“I request permission for the open-access journal PLOS ONE to publish XXX under the Creative Commons Attribution License (CCAL) CC BY 4.0 (http://creativecommons.org/licenses/by/4.0/). Please be aware that this license allows unrestricted use and distribution, even commercially, by third parties. Please reply and provide explicit written permission to publish XXX under a CC BY license and complete the attached form.”

Please upload the completed Content Permission Form or other proof of granted permissions as an "Other" file with your submission.

In the figure caption of the copyrighted figure, please include the following text: “Reprinted from [ref] under a CC BY license, with permission from [name of publisher], original copyright [original copyright year].”

b. If you are unable to obtain permission from the original copyright holder to publish these figures under the CC BY 4.0 license or if the copyright holder’s requirements are incompatible with the CC BY 4.0 license, please either i) remove the figure or ii) supply a replacement figure that complies with the CC BY 4.0 license. Please check copyright information on all replacement figures and update the figure caption with source information. If applicable, please specify in the figure caption text when a figure is similar but not identical to the original image and is therefore for illustrative purposes only.

8. Please ensure that you refer to Figure 9, 11, 12 in your text as, if accepted, production will need this reference to link the reader to the figure.

9. We note that Figure [6, 8, 13] includes an image of a [patient / participant / in the study].

As per the PLOS ONE policy (http://journals.plos.org/plosone/s/submission-guidelines#loc-human-subjects-research) on papers that include identifying, or potentially identifying, information, the individual(s) or parent(s)/guardian(s) must be informed of the terms of the PLOS open-access (CC-BY) license and provide specific permission for publication of these details under the terms of this license. Please download the Consent Form for Publication in a PLOS Journal (http://journals.plos.org/plosone/s/file?id=8ce6/plos-consent-form-english.pdf). The signed consent form should not be submitted with the manuscript, but should be securely filed in the individual's case notes. Please amend the methods section and ethics statement of the manuscript to explicitly state that the patient/participant has provided consent for publication: “The individual in this manuscript has given written informed consent (as outlined in PLOS consent form) to publish these case details”.

If you are unable to obtain consent from the subject of the photograph, you will need to remove the figure and any other textual identifying information or case descriptions for this individual."

10. We note you have included a table to which you do not refer in the text of your manuscript. Please ensure that you refer to Table 1, 2 in your text; if accepted, production will need this reference to link the reader to the Table.

11. If the reviewer comments include a recommendation to cite specific previously published works, please review and evaluate these publications to determine whether they are relevant and should be cited. There is no requirement to cite these works unless the editor has indicated otherwise.

Additional Editor Comments:

Please revise as per comments.

[Note: HTML markup is below. Please do not edit.]

Reviewers' comments:

Reviewer's Responses to Questions

**Comments to the Author**

1. Is the manuscript technically sound, and do the data support the conclusions?

Reviewer #1: Partly

Reviewer #2: Yes

**********

2. Has the statistical analysis been performed appropriately and rigorously?

Reviewer #1: No

Reviewer #2: N/A

**********

3. Have the authors made all data underlying the findings in their manuscript fully available?

The PLOS Data policy

Reviewer #1: No

Reviewer #2: Yes

**********

4. Is the manuscript presented in an intelligible fashion and written in standard English?

Reviewer #1: Yes

Reviewer #2: Yes

**********

Reviewer #1: 1) The proposed method primarily combines:

a) well-known YOLOv5 architectural modifications for small object detection,

b) patch-based inference (tiling),

c) heuristic color-based preprocessing,

d) a manually designed fuzzy inference system.

Each of these components has been previously explored in the literature and the manuscript does not clearly

demonstrate what fundamentally new methodological insight is introduced beyond system integration. The

novelty appears incremental rather than conceptual. Explicitly clarify the unique contribution.

2) The approach relies heavily on Cr-channel color probability maps under the assumption that

1. Cr-channel clusters reliably correspond to faces,

2. cluster density correlates with face distance,

3. skin-tone representation is stable across lighting, ethnicity and cameras.

These assumptions are not rigorously validated and raise concerns regarding robustness and bias.

3) The evaluation protocol has several critical limitations:

a) The proposed dataset is small, non-public and lacks sufficient statistical diversity.

b) Comparisons with recent detectors (e.g., YOLOv8/YOLO-NAS small-object variants) are missing.

c) Real-time claims are not supported by inference speed or computational cost analysis.

4. The introduction is overly verbose and contains generic AI background; it should be significantly

condensed.

5. Several figures (e.g., color maps and heatmaps) are qualitative and should be supported by

quantitative analysis.

6. Terminology such as “accuracy” is used inconsistently instead of AP or mAP.

7. Dataset statistics in Table 1 contain inconsistencies and require clarification.

8. Grammar and stylistic issues are present throughout and require careful proofreading.

Reviewer #2: This study presents a noteworthy methodology for detecting small faces in images by integrating deep learning and fuzzy logic. Nevertheless, several concerns require attention before the work can be fully evaluated

Comments

•In the Abstract and Introduction section, the manuscript does not clearly establish its novelty. While multiple contributions are listed, it remains unclear what is fundamentally new compared to existing works. The paper lacks a strong positioning of its contribution as a unified system-level innovation, which weakens its impact.

•In the Methodology section, specifically the Fuzzy Inference System, the justification for using fuzzy logic is insufficient. The manuscript introduces rule-based decisions but does not explain why fuzzy logic is preferred over simpler thresholding or modern learning-based approaches, nor does it provide a comparative analysis.

•In the Color Probability Maps section, the assumption that the Cr channel effectively represents human skin is not rigorously justified. This approach may not generalize well across varying lighting conditions, skin tones, or environmental scenarios, and the manuscript lacks discussion of these limitations.

•In the Results section, the comparison with state-of-the-art methods is limited. The manuscript does not include recent models such as newer YOLO variants or transformer-based detectors, and it is unclear whether the comparisons are conducted under fair and consistent experimental settings.

•In the Custom Dataset description, the dataset design is not sufficiently justified. A large portion of images lack face annotations, which may weaken the reliability of the evaluation. The manuscript does not adequately explain how this affects performance assessment.

•Across the Methodology and Results sections, the manuscript lacks an ablation study. There is no clear breakdown of how each component (modified architecture, patching strategy, fuzzy system) contributes individually to the final performance, making it difficult to assess the effectiveness of each module.

oIn the Introduction section, the writing style includes informal phrases such as 'in our opinion,' which are not suitable for academic writing. A more formal tone should be adopted throughout the manuscript.

oThroughout the manuscript, there are multiple grammatical and sentence structure inconsistencies. These issues affect readability and clarity, and the paper would benefit from thorough proofreading or professional editing.

oIn several figures, particularly Figure 11, the explanations are insufficient. The captions and in-text descriptions do not clearly guide the reader in interpreting the results, which reduces the effectiveness of the visual content.

oIn the Evaluation Metrics section, the equations are not consistently formatted. Some equations lack proper numbering or alignment, which makes them harder to follow.

oIn the References section, there are repeated references (Ref 12 & 15). The manuscript would benefit from including more recent and diverse references to better position the work within current research.

oIn Table 1, the formatting and values appear inconsistent and unclear. The table should be revised to ensure the information is presented accurately and easily interpreted.

**********

what does this mean?). If published, this will include your full peer review and any attached files.

If you choose “no”, your identity will remain anonymous but your review may still be made public.

**Do you want your identity to be public for this peer review?**  For information about this choice, including consent withdrawal, please see our Privacy Policy

Reviewer #1: **Yes:**  Rana Sami Ullah Khan

Reviewer #2: No

**********

[NOTE: If reviewer comments were submitted as an attachment file, they will be attached to this email and accessible via the submission site. Please log into your account, locate the manuscript record, and check for the action link "View Attachments". If this link does not appear, there are no attachment files.]

To ensure your figures meet our technical requirements, please review our figure guidelines: https://journals.plos.org/plosone/s/figures

You may also use PLOS’s free figure tool, NAAS, to help you prepare publication quality figures: https://journals.plos.org/plosone/s/figures#loc-tools-for-figure-preparation.

NAAS will assess whether your figures meet our technical requirements by comparing each figure against our figure specifications.

Attachment Submitted filename: manuscript comments.pdf

# Author comment: Author response to Decision Letter 1

**DOI:** 10.1371/journal.pone.0360794.r002

11 Aug 2026

Response to the Academic Editor and Reviewers

Manuscript ID: PONE-D-25-65594

YOLO-SOD: Color probability map-based fuzzy framework using deep models for face detection

Dear Academic Editor and Reviewers,

We sincerely thank the Academic Editor and both reviewers for their careful assessment and constructive comments. We have extensively revised the manuscript in light of the feedback. The revision clarifies the system level novelty, expands quantitative validation of the Cr-channel cues, explains the choice of fuzzy inference, adds direct baseline comparisons, and component wise ablation studies, clarifies the local dataset design and split, standardizes the evaluation terminology and equations, and improves the figures, tables, references, and overall presentation. The detailed point-by-point responses are provided below. Page and line references correspond to the revised clean manuscript dated August 09, 2026.

Evaluation clarification and regenerated graphs

As part of the revision, we checked the evaluation pipeline and identified an implementation inconsistency in the earlier AP calculation. The affected values and plots were recomputed and the corresponding Wider Face and local dataset graphs, tables, Abstract, Results, and Conclusion were updated. For WIDER FACE, evaluation uses the official Easy/Medium/Hard .mat annotations and the standard 1,000-threshold AP protocol at IoU 0.50 through a Python implementation of the official MATLAB evaluator; comparison graphs load the official baseline .mat precision-recall curves. For the local dataset, a common VOC/YOLO-style AP@0.50 evaluator is applied identically to YOLOv5, YOLOv8, and YOLO-SOD using confidence ranked detections, one-to-one ground-truth matching, and precision-recall curve integration. This standardization ensures that all reported comparisons use consistent evaluation rules.

A. Response to the Academic Editor and Journal Requirements

Comment Response and location of revision

Journal requirement 1

Ensure that the manuscript meets PLOS ONE style and file-naming requirements. The manuscript has been reformatted using the current PLOS ONE LaTeX template.

Location in revised manuscript: Formatting changes apply throughout the manuscript.

Journal requirement 2

Author-generated code underpinning the findings must be shared without restriction and in a reproducible form. The code has been uploaded and shared at GITHUB link https://github.com/YawarGuguma/color-probability-maps

Journal requirements 3–4

Clarify restrictions on data sharing or deposit the minimal anonymized dataset in a stable public repository. Dataset has been uploaded here

https://doi.org/10.6084/m9.figshare.33189120

Password is 404

Journal requirement 5

Funding information and the financial disclosure do not match; provide the correct grant information. This is the PhD study and supported through NED research fund. The acknowledgement has been included inside the paper.

Journal requirement 6

State the role of the funder. Funder: NED University of Engineering and Technology (NEDUET)

NEDUET supported this study because the scholar is a PhD student at NEDUET and striving to complete her PhD.

Journal requirement 7

Figures 1, 2, 3, 4, 5, 6, 7, 8, 10, and 13 were flagged for possible copyrighted material. Emails were sent to take permission but did not received the reply.

Therefore, the copyrighted material has been removed please. The datasets are public and the reader may download and see the dataset images

Journal requirement 8

Refer to Figures 9, 11, and 12 in the manuscript text. Authors want to thank the editor for this comment. All Figures have been cited please.

Journal requirement 9

Figures containing identifiable participants require specific written consent for publication under the PLOS CC BY license. We acknowledge the journal’s participant consent requirement. Identifiable participant images will be retained only where the required PLOS publication consent has been obtained and securely filed. The ethics/methods statement will explicitly state that written informed consent for publication under the PLOS terms was obtained. Where consent is unavailable, the relevant image will be removed or replaced with a non-identifying visualization or blurring.

Journal requirement 10

Refer to Tables 1 and 2 in the manuscript text. Both tables are now cited in the body text. Thank you.

Journal requirement 11

Evaluate reviewer-recommended literature and cite it only when relevant. Acknowledged and noted. No references were suggested by the reviewers. Thank you.

Additional Editor Comment

Please revise as per comments. The manuscript has been comprehensively revised in response to all journal requirements and reviewer comments. The specific revisions are itemized below.

B. Response to Reviewer 1 (p is page; pp is pages)

Comment Response and location of revision

Reviewer 1, Comment 1

The novelty appears incremental because the method combines known YOLO modifications, tiling, color preprocessing, and fuzzy inference. Clarify the unique contribution. We agree that the novelty needed clearer positioning. The revised manuscript now presents the contribution as a unified system level solution rather than claiming that each component is individually. The distinctive contribution is the joint use of (i) higher resolution YOLOv5 feature pathways and small object detection heads and (ii) image-adaptive patch configuration inferred from Cr cluster count and scale cues through a lightweight FIS. Unlike fixed tiling, the number and size of patches are selected separately for each image. Component wise ablations were also added to quantify the contribution of the architecture and adaptive fuzzy patching.

Location in revised manuscript: Abstract, p. 1; Introduction, pp. 2–3, lines 27–65; Problem identification, p. 5, lines 174–192; WIDER ablation, Table 7; local ablation, Table 10.

Reviewer 1, Comment 2

The Cr-channel assumptions are insufficiently validated: clusters may not correspond to faces, density may not correlate with distance, and representation may vary across lighting, ethnicity, and cameras. We substantially expanded this analysis. First, a head region contrast score was computed across RGB, HSV, and YCbCr channels using 500 aligned WIDER FACE crops; Cr achieved the highest Head region Contrast Score (1.818). Second, an image level analysis on 136 WIDER FACE images (1,310 faces) showed that images with more annotated faces generally produced more Cr candidate clusters, while explicitly stating that not every cluster is a face. Third, face scale groups were defined by normalized bounding box height, and Cr cluster size increased across small, medium, and large face groups. The manuscript now clearly states that cluster count and density are coarse patch selection cues, not direct estimates of face count or physical distance. A limitation statement also clarifies that invariance across skin tone, illumination, and camera characteristics is not established.

Location in revised manuscript: Cr channel analysis, pp. 11–15, lines 319–420; Fig. 5; Tables 1–3; limitation statement on p. 13, lines 366–368.

Reviewer 1, Comment 3(a)

The proposed dataset is small, non-public, and lacks sufficient statistical diversity. The revised manuscript now gives complete dataset statistics, positive and negative image counts, annotated face counts, and separate train/validation/test splits. The rationale for retaining background only images is also explained: they reflect realistic dashcam conditions and test false positive suppression when no face is present. The limitation associated with dataset scale is acknowledged through the more cautious interpretation of the local results.

Location in revised manuscript: Proposed dataset, pp. 19–20, lines 464–490; Tables 4 and 5; local test rationale, p. 24, lines 555–564.

Reviewer 1, Comment 3(b)

Comparisons with recent detectors, including newer YOLO variants, are missing. We added a controlled comparison with standard YOLOv5 and YOLOv8 on both WIDER FACE and the local proposed dataset.

Location in revised manuscript: Table 6, pp. 23–24; local comparison, Table 9, p. 24.

Reviewer 1, Comment 3(c)

Real-time claims are unsupported by inference-speed or computational-cost analysis. We have avoided presenting YOLO-SOD as experimentally verified to be real-time. References to real-time behavior now describe previously published baseline architectures rather than a measured claim for the proposed method. The revised experimental discussion focuses on mAP50, uses Nano YOLO variants for a controlled comparison, and describes computational considerations qualitatively without reporting FPS claims.

Location in revised manuscript: Local results, p. 24, lines 568–575.

Reviewer 1, Comment 4

The Introduction is overly verbose and contains generic AI background. The Introduction was condensed and reorganized around the specific small face detection problem.

Location in revised manuscript: Introduction, pp. 2–3, lines 1–68.

Reviewer 1, Comment 5

The qualitative color maps and heatmaps should be supported by quantitative analysis. Quantitative validation was added. The revised manuscript reports channel wise Head region Contrast Score values, image level Cr cluster abundance statistics, face level cluster size statistics, and the proportion of annotated faces associated with at least one candidate cluster. These analyses directly support and bound the interpretation of Figures 5 and 6.

Location in revised manuscript: pp. 12–15, lines 352–420; Tables 1–3.

Reviewer 1, Comment 6

Terminology such as “accuracy” is used inconsistently instead of AP or mAP. The primary metric is now explicitly defined as mAP50. General uses of the word “accuracy” are intended only as broad meaning and not as a separate metric.

Location in revised manuscript: Evaluation Metrics, p. 22, lines 496–527; Tables 6–11; Fig. 11.

Reviewer 1, Comment 7

Dataset statistics in Table 1 are inconsistent and require clarification. The former dataset statistics presentation was corrected and expanded. The revised manuscript now separates overall dataset information from split-level statistics. Thank you

Location in revised manuscript: Tables 4 and 5, pp. 19–20, lines 471–490.

Reviewer 1, Comment 8

Grammar and stylistic issues require careful proofreading. The manuscript was extensively revised and edited for grammar and sentence structure.

Location in revised manuscript: Changes throughout the manuscript.

Evaluation recalculation

The evaluation protocol and result reliability require clarification. During the revision, an implementation inconsistency in the earlier mAP50 calculation was corrected and all affected results and plots were regenerated. WIDER FACE now uses official Easy/Medium/Hard .mat annotations and the standard 1,000-threshold protocol at IoU 0.50 through a Python implementation of the official MATLAB evaluator. The local dataset uses the same confidence ranked, one-to-one matching, PR-integration evaluator for YOLOv5, YOLOv8, and YOLO-SOD. The corrected WIDER FACE values are 93.07%, 91.25%, and 84.45% for Easy, Medium, and Hard; the corrected local YOLO-SOD value is 49.36%.

Location in revised manuscript: Abstract, p. 1; Evaluation Metrics, p. 22; WIDER FACE results, pp. 23–24, Fig. 11 and Tables 6–7; local results, pp. 24–25, Tables 9–10; Conclusion, p. 26.

C. Response to Reviewer 2

Comment Response and location of revision

Reviewer 2, Comment 1

The Abstract and Introduction do not clearly establish novelty or position the work as a unified system-level contribution. The Abstract and Introduction were rewritten to frame the work as a unified small-face detection framework. The revised positioning emphasizes the interaction between high resolution detection pathways from YOLO backbone, neck, and head to image adaptive fuzzy patch selection. The unique contribution is also stated explicitly in the Problem identification subsection and supported by ablation studies.

Location in revised manuscript: Abstract, p. 1; Introduction, pp. 2–3, lines 27–65; Problem identification, p. 5, lines 174–192; Tables 7 and 10.

Reviewer 2, Comment 2

The manuscript does not justify why fuzzy logic is preferred over hard thresholds or a learned selection model. A dedicated justification was added. The revised text explains that fixed threshold ranges require extensive manual tuning, whereas an additional learned model would introduce extra complexity and supervision solely for patch selection. The Fuzzy system provides a lightweight way to combine cluster count and density through overlapping membership functions and interpretable rules.

Location in revised manuscript: Fuzzy patches subsection, p. 16, lines 421–429, followed by the five fuzzy rules at lines 439–444.

Reviewer 2, Comment 3

The Cr channel may not generalize across lighting, skin tones, or environmental conditions, and the limitations are not discussed. The revised manuscript adds quantitative support while explicitly limiting the claim. Cr is treated only as a heuristic scene level cue for patch selection, not as a skin classifier or face detector. The text states that WIDER FACE lacks explicit metadata for skin tone, illumination, and camera characteristics, and therefore invariance across these factors is not established.

Location in revised manuscript: Cr channel section, pp. 11–15, especially p. 13, lines 366–368, and pp. 14–15, lines 372–420.

Reviewer 2, Comment 4

The Results comparison is limited and the fairness of comparisons is unclear. We added comparisons with YOLOv5 and YOLOv8, using consistent model scale and evaluation procedures. WIDER FACE results are reported on the official Easy, Medium, and Hard validation subsets. Figure 11 uses official baseline .mat precision-recall curves for literature methods, while Tables 6 and 9 distinguish the directly evaluated baselines.

Location in revised manuscript: WIDER results, pp. 23–24; local results, p. 24.

Reviewer 2, Comment 5

The custom dataset design is insufficiently justified, particularly the large number of images without faces. The revised manuscript explains that background-only images were intentionally retained to reproduce real dashcam conditions, where faces are sparse or absent. These images test whether the detector suppresses false positives, while positive images evaluate small and distant face detection. The complete positive/negative distribution and split statistics are now reported.

Location in revised manuscript: Dataset description, pp. 19–20, lines 471–490; local-results rationale, p. 24, lines 555–564; Tables 4 and 5.

Reviewer 2, Comment 6

The manuscript lacks an ablation study for the modified architecture, patching strategy, and fuzzy system. Two component wise ablation studies were added. Table 7 reports the WIDER FACE progression from baseline YOLOv5 to the modified architecture and then to fuzzy Cr patches. Table 10 reports the same progression on the local dataset.

Location in revised manuscript: Table 7, p. 24, lines 552–554; Table 10, p. 25, lines 576–579.

Reviewer 2, Comment 7

Informal phrases such as “in our opinion” should be replaced by formal academic wording. The manuscript has been substantially revised toward a more formal tone

Reviewer 2, Comment 8

Grammatical and sentence-structure inconsistencies affect readability. The manuscript was extensively proofread and edited for grammar, sentence structure, and clarity.

Location in revised manuscript: Changes throughout the manuscript.

Reviewer 2, Comment 9

Figure 11 and several other figures require clearer captions and in-text explanations. Figure 11 was replaced with three clearly labeled precision-recall panels for the Easy, Medium, and Hard subsets. All other figures were reviewed and their caption were modified where necessary

Location in revised manuscript: Figure 11 and WIDER Results, p. 23, lines 541–547; Figures 5–6 and supporting Tables 1–3, pp. 12–15.

Reviewer 2, Comment 10

Equations in the Evaluation Metrics section are inconsistently formatted or numbered. The evaluation equations were reformatted as numbered display equations and explained immediately after presentation. The revised section defines mAP50, precision, recall, and PR-curve integration in a consistent sequence.

Location in revised manuscript: Evaluation Metrics, p. 22, lines 496–527; Equations 4–7.

Reviewer 2, Comment 11

The References contain a duplicate and should include more recent and diverse work. The duplicate reference was removed and the bibliography was checked for unique entries.

Location in revised manuscript: Related Work, pp. 3–5; References, pp. 26–30.

Reviewer 2, Comment 12

Table 1 formatting and values are inconsistent and unclear. The tables were reorganized and clarified. The revised Table 1 now reports a clearly defined quantitative Head region Contrast Score analysis.

The former dataset statistics were corrected and expanded into Tables 4 and 5 so that totals and split-level values are transparent and internally consistent.

Location in revised manuscript: Table 1, p. 13; Tables 4–5, pp. 19–20.

Corrected WIDER/local graphs

The revised result plots should be based on a consistent and clearly stated evaluation procedure. The WIDER FACE and local result graphs were regenerated after standardizing the metric implementation. WIDER FACE uses official subset annotations and the standard 1,000-threshold evaluator at IoU 0.50, and the local comparison uses one identical evaluator for all three methods. The revised values are consistently propagated to the Abstract, result tables, Figure 11, and Conclusion.

Location in revised manuscript: Abstract, p. 1; Evaluation Metrics, p. 22; WIDER Results, pp. 23–24; local Results, pp. 24–25; Conclusion, p. 26.

Sincerely,

Ms. Aniqa Saba,

Prof. Dr. Muhammad Faizan Shirazi,

Prof. Dr. Yawar Rehman,

Prof. Dr. Muhammad Mubashir Khan

Attachment Submitted filename: Response_to_Editor_and_Reviewers_PONE-D-25-65594_modified.docx

# Peer review: Decision Letter 1

**DOI:** 10.1371/journal.pone.0360794.r003

**Authors:** Usman Ghafoor

17 Sep 2026

Dear Dr. Shirazi,

Thank you for submitting your manuscript to PLOS One. After careful consideration, we feel that it has merit but does not fully meet PLOS One’s publication criteria as it currently stands. Therefore, we invite you to submit a revised version of the manuscript that addresses the points raised during the review process.

==============================

**ACADEMIC EDITOR:**  Please critically resolve these minor issues.

==============================

Please submit your revised manuscript by Nov 01 2026 11:59PM. If you will need more time than this to complete your revisions, please reply to this message or contact the journal office at plosone@plos.org. When you're ready to submit your revision, log on to https://www.editorialmanager.com/pone/ and select the 'Submissions Needing Revision' folder to locate your manuscript file.

- A letter that responds to each point raised by the academic editor and reviewer(s). You should upload this letter as a separate file labeled 'Response to Reviewers'.
- A marked-up copy of your manuscript that highlights changes made to the original version. You should upload this as a separate file labeled 'Revised Manuscript with Track Changes'.
- An unmarked version of your revised paper without tracked changes. You should upload this as a separate file labeled 'Manuscript'.

If you would like to make changes to your financial disclosure, please include your updated statement in your cover letter. Guidelines for resubmitting your figure files are available below the reviewer comments at the end of this letter.

If applicable, we recommend that you deposit your laboratory protocols in protocols.io to enhance the reproducibility of your results. Protocols.io assigns your protocol its own identifier (DOI) so that it can be cited independently in the future. For instructions see: https://journals.plos.org/plosone/s/submission-guidelines#loc-laboratory-protocols. Additionally, PLOS One offers an option for publishing peer-reviewed Lab Protocol articles, which describe protocols hosted on protocols.io. Read more information on sharing protocols at https://plos.org/protocols?utm_medium=editorial-email&utm_source=authorletters&utm_campaign=protocols.

As the corresponding author, your ORCID iD is verified in the submission system and will appear in the published article. PLOS supports the use of ORCID, and we encourage all coauthors to register for an ORCID iD and use it as well. Please encourage your coauthors to verify their ORCID iD within the submission system before final acceptance, as unverified ORCID iDs will not appear in the published article. *Only*  the individual author can complete the verification step; PLOS staff *cannot*  verify ORCID iDs on behalf of authors.

We look forward to receiving your revised manuscript.

Kind regards,

Usman Ghafoor

Academic Editor

PLOS One

Journal Requirements:

If the reviewer comments include a recommendation to cite specific previously published works, please review and evaluate these publications to determine whether they are relevant and should be cited. There is no requirement to cite these works unless the editor has indicated otherwise.

Please review your reference list to ensure that it is complete and correct. If you have cited papers that have been retracted, please include the rationale for doing so in the manuscript text, or remove these references and replace them with relevant current references. Any changes to the reference list should be mentioned in the rebuttal letter that accompanies your revised manuscript. If you need to cite a retracted article, indicate the article’s retracted status in the References list and also include a citation and full reference for the retraction notice.

Additional Editor Comments :

Please critically resolve these minor issues.

[Note: HTML markup is below. Please do not edit.]

Reviewers' comments:

Reviewer's Responses to Questions

**Comments to the Author**

Reviewer #1: (No Response)

Reviewer #2: (No Response)

**********

2. Is the manuscript technically sound, and do the data support the conclusions?

Reviewer #1: (No Response)

Reviewer #2: Yes

**********

3. Has the statistical analysis been performed appropriately and rigorously?

Reviewer #1: (No Response)

Reviewer #2: Yes

**********

4. Have the authors made all data underlying the findings in their manuscript fully available?

The PLOS Data policy

Reviewer #1: (No Response)

Reviewer #2: Yes

**********

5. Is the manuscript presented in an intelligible fashion and written in standard English?

Reviewer #1: (No Response)

Reviewer #2: Yes

**********

Reviewer #1: Minor revisions recommended

1. Correct the Wider Face result inconsistency.

The Abstract reports 83.4%, 84.8%, and 70.4% for Easy/Medium/Hard, while the Results section reports 83.4%, 84.4%, and 70.4%. Choose the verified value and use it consistently throughout the Abstract, Results, tables, and Conclusion.

2. Correct Table 1.

There appears to be a serious formatting/numerical corruption in the dataset table. The text says there are 436 images and 350 face annotations, but Table 1 appears as “35024,” “545,” and “88,” which is clearly inconsistent with the surrounding description.

Verify and reconstruct the table from the original dataset records before resubmission.

3. Clarify the annotation counts.

The manuscript states:

o 436 total images

o 271 images with no faces

o 83 images with one face

o 67 images with 2–4 faces

o 12 images with 5–7 faces

o 3 images with 8–10 faces

These image counts sum to 436, which is good, but the stated 350 annotations should be reconciled mathematically with these categories. Add a short explanation of exactly how the 350 annotations were calculated.

4. Use consistent terminology throughout.

Standardize:

o YOLOv5 rather than alternating between Yolo, Yolo5, YOLO5, and YOLO v5

o WIDER FACE rather than Wider Face/Wider dataset

o VisDrone-DET2019 consistently

o mAP@50 or AP50 consistently

o “color probability map” consistently

o “patch size” rather than occasionally “path size” or “patches size.”

5. Correct the title terminology.

The title currently says:

“YOLOv5-Small Object Detection: Color Probability Map Based Fuzzy Framework Using Deep Models for Face Detection”

A cleaner version would be:

“YOLO-SOD: A Color-Probability-Map-Based Fuzzy Framework for Small-Face Detection Using YOLOv5”

This better reflects the actual contribution.

6. Improve the description of the YOLOv5 architecture.

The manuscript says the default YOLOv5 has three heads and later says the proposed model has four heads, while also describing removal/addition of heads. This should be presented in a concise table:

Component Original YOLOv5 Proposed YOLO-SOD

Detection heads 3 4

Feature maps 80×80, 40×40, 20×20 320×320, 160×160, 80×80, 40×40

Added C3 blocks — 4

20×20 head Present Removed

7. The current architectural description supports this information but makes the reader reconstruct it themselves.

8. Explain the fuzzy membership functions quantitatively.

The five fuzzy rules are given, but the manuscript does not adequately specify the membership functions/ranges for “low,” “medium,” and “high” cluster number/density.

Add a small table specifying:

o input variables,

o membership ranges,

o membership-function type,

o output patch sizes,

o defuzzification method.

9. Tone down unsupported statements.

For example:

“current state-of-the-art deep models ... may fail to detect the faces in the wild”

and:

“conventional state-of-the-art networks often fail.”

These are stronger than necessary. Replace with evidence-based wording such as:

“Performance may decrease under unconstrained conditions involving small face size, occlusion, pose variation, and complex backgrounds.”

The manuscript itself identifies these conditions as challenges.

10. Replace subjective wording.

Phrases such as:

o “In our opinion”

o “One sentence answer is…”

o “very good clues”

o “golden 80% accuracy mark”

o “intelligent, cluster-aware modification”

o “promising transfer”

should be changed to objective scientific language. For example, “golden 80% accuracy mark” should simply be removed.

11. Strengthen the limitations section.

The manuscript acknowledges that far-distance faces were excluded because of poor image quality. This should explicitly appear in the Limitations section, together with:

o dependence of the Cr-based map on illumination/skin-color characteristics,

o possible false clusters caused by background regions,

o limited size of the proposed local dataset,

o lack of evaluation for extremely distant faces.

12. Clarify the local dataset methodology.

Include, if available:

o where/how images were collected,

o collection period,

o camera/device information,

o selection criteria,

o annotation procedure,

o annotator verification,

o whether the dataset is publicly available.

This is particularly important because the local dataset is presented as a contribution.

13. Improve the evaluation description.

The manuscript explains mAP/AP at considerable length, but some of the discussion is unnecessarily textbook-like. For a research article, this can be shortened and replaced with the exact evaluation protocol used in the experiments.

14. Add an ablation study if the experimental data are available.

This would substantially strengthen the paper without changing the methodology. At minimum:

o baseline YOLOv5

o YOLOv5 + modified architecture

o YOLOv5 + fixed patching

o YOLOv5 + fuzzy patch selection

o complete YOLO-SOD

This would demonstrate whether the improvement comes from the architectural modification, the patching, the fuzzy system, or their combination.

15. Correct grammar throughout.

There are many recurring grammatical problems, for example:

“These feature maps works perfect...”

→ “These feature maps work well...”

“the detection head ... get the feature map”

→ “the detection head ... receives the feature map”

“We observed that the accuracy...”

→ “We observed that detector accuracy...”

“faces at near to medium distance”

→ “faces at near-to-medium distances”

“patches plays an important”

→ “patches play an important role”

16. Correct the Conclusion formatting.

The Conclusion currently contains an apparent stray word:

“Use This work improves...”

This should be removed. Also insert spaces between the reported values:

“83.4%84.8%70.4 AP”

should be:

“83.4%, 84.8%, and 70.4% AP”.

Highest-priority fixes

If you want this to remain a minor revision, I would prioritize these five:

1. Fix Table 1 and reconcile the dataset numbers.

2. Resolve 84.8% vs 84.4% for WIDER FACE.

3. Standardize YOLOv5/WIDER FACE/VisDrone/mAP terminology.

4. Add the fuzzy membership-function details.

5. Perform a thorough English/grammar and scientific-style edit.

The underlying contribution—modified YOLOv5 architecture + adaptive patching + Cr-based clustering + FIS—is already clearly described in the manuscript. I would not make major changes to the proposed method unless the reviewer specifically requests additional experiments.

Reviewer #2: Thank you for the careful revision. I have read the revised manuscript together with the point-by-point response. In my view, the authors have addressed the main concerns raised in the previous round. At this stage, I do not think another major experimental revision is necessary. The remaining issues are mainly about wording, interpretation, and presentation, and they should be straightforward to address in a minor revision.

Minor comments

1. Please use AP/mAP terminology consistently throughout the manuscript. In particular, Table 9 is titled “Test accuracy” although the reported metric is mAP50, and there are still sentences that describe mAP50 values as “accuracy.” These should be revised simply to report the results as mAP50.

2. The interpretation of the ablation results should be slightly moderated. The modified YOLOv5 architecture provides the dominant improvement, while the additional fuzzy Cr-based patching stage gives a smaller incremental gain. Therefore, statements such as “each module improves the accuracy” should be rephrased so that they accurately reflect the values in Tables 7 and 10. I do not consider additional experiments essential here; a more precise discussion of the reported results would be sufficient.

3. A final careful language edit is still recommended. There are several remaining grammatical and typographical issues (for example, expressions such as “fastly trainable,” “the background strengthen,” and similar wording). Cleaning these up would improve the readability and presentation of the final manuscript.

**********

what does this mean?). If published, this will include your full peer review and any attached files.

If you choose “no”, your identity will remain anonymous but your review may still be made public.

**Do you want your identity to be public for this peer review?**  For information about this choice, including consent withdrawal, please see our Privacy Policy

Reviewer #1: No

Reviewer #2: No

**********

[NOTE: If reviewer comments were submitted as an attachment file, they will be attached to this email and accessible via the submission site. Please log into your account, locate the manuscript record, and check for the action link "View Attachments". If this link does not appear, there are no attachment files.]

To ensure your figures meet our technical requirements, please review our figure guidelines: https://journals.plos.org/plosone/s/figures

You may also use PLOS’s free figure tool, NAAS, to help you prepare publication quality figures: https://journals.plos.org/plosone/s/figures#loc-tools-for-figure-preparation.

NAAS will assess whether your figures meet our technical requirements by comparing each figure against our figure specifications.

# Author comment: Author response to Decision Letter 2

**DOI:** 10.1371/journal.pone.0360794.r004

30 Sep 2026

Response to the Academic Editor and Reviewers

Manuscript ID: PONE-D-25-65594R1

YOLO-SOD: Color probability map-based fuzzy framework using deep models for face detection

Dear Academic Editor and Reviewers,

We thank you for your valuable comment for improving our paper. Following we present point by point response and the exact location of each revision.

Additional comments to Editor

It is to inform that the affiliation of author Yawar Rehman’s has been updated. Thank you.

A. Response to the Academic Editor and Journal Requirements

Comment Response and location of revision

Academic Editor Comment

Please critically resolve these minor issues. Authors thank the editor and all the reviewers for their efforts for the improvement of our paper. We have critically analyzed and resolved the highlighted minor issues.

Journal requirement - References

Please review your reference list to ensure that it is complete and correct. If you have cited papers that have been retracted, please include the rationale for doing so in the manuscript text, or remove these references and replace them with relevant current references. Any changes to the reference list should be mentioned in the rebuttal letter that accompanies your revised manuscript. If you need to cite a retracted article, indicate the article's retracted status in the References list and also include a citation and full reference for the retraction notice. Thank you for this comment. We carefully reviewed the complete reference list. None of the reference is found to be retracted.

B. Response to Reviewer 1

Comment Response and location of revision

Reviewer 1, Comment 1

Correct the WIDER FACE result inconsistency.

The Abstract reports 83.4%, 84.8%, and 70.4% for Easy/Medium/Hard, while the Results section reports 83.4%, 84.4%, and 70.4%. Choose the verified value and use it consistently throughout the Abstract, Results, tables, and Conclusion. We think there has been some confusion because the results reported in the submitted revision 1 for Wider Face dataset are 93.07% mAP50 (Easy), 91.25% mAP50 (Medium), and 84.45% mAP50 (Hard) are consistent throughout the manuscript.

Reviewer 1, Comment 2

Correct Table 1.

There appears to be a serious formatting/numerical corruption in the dataset table. The text says there are 436 images and 350 face annotations, but Table 1 appears as "35024," "545," and "88," which is clearly inconsistent with the surrounding description.

Verify and reconstruct the table from the original dataset records before resubmission. We think there has been some confusion because Table 1 in the submitted revision 1 represents contrast score analysis for different datasets.

And the values that the reviewer mentioned in this comment pertains to Table 4 for submitted revision 1, which is accurate with its surrounding explanation.

Reviewer 1, Comment 3

Clarify the annotation counts.

The manuscript states:

- 436 total images

- 271 images with no faces

- 83 images with one face

- 67 images with 2-4 faces

- 12 images with 5-7 faces

- 3 images with 8-10 faces

These image counts sum to 436, which is good, but the stated 350 annotations should be reconciled mathematically with these categories. Add a short explanation of exactly how the 350 annotations were calculated. Table 8: Distribution of images and face annotations in the proposed dataset, has been included to reconcile the 436 image with 350 annotations.

Reviewer 1, Comment 4

Use consistent terminology throughout. Standardize:

- YOLOv5 rather than alternating between Yolo, Yolo5, YOLO5, and YOLO v5

- WIDER FACE rather than Wider Face/Wider dataset

- VisDrone-DET2019 consistently

- mAP@50 or AP50 consistently

- "color probability map" consistently

- "patch size" rather than occasionally "path size" or "patches size." We thank you reviewer for this comment. All the terminologies in the manuscript has been made consistent as suggest by the reviewer.

Reviewer 1, Comment 5

Correct the title terminology.

The title currently says: "YOLOv5-Small Object Detection: Color Probability Map Based Fuzzy Framework Using Deep Models for Face Detection"

A cleaner version would be: "YOLO-SOD: A Color-Probability-Map-Based Fuzzy Framework for Small-Face Detection Using YOLOv5"

This better reflects the actual contribution. We thank reviewer for this comment. However, this paper is related to the approved PhD title from ASRB board of NED university, changing the title of the paper would complicate things for the PhD degree please. We sincerely hope and request understanding on this matter. Thank you.

Our current title of the paper is as follows as submitted in the revision 1.

“YOLO-SOD: Color probability map-based fuzzy framework using deep models for face detection”

Reviewer 1, Comment 6

Improve the description of the YOLOv5 architecture.

The manuscript says the default YOLOv5 has three heads and later says the proposed model has four heads, while also describing removal/addition of heads. This should be presented in a concise table:

Component | Original YOLOv5 | Proposed YOLO-SOD

Detection heads | 3 | 4

Feature maps | 80x80, 40x40, 20x20 | 320x320, 160x160, 80x80, 40x40

Added C3 blocks | - | 4

20x20 head | Present | Removed Table 1: Comparison of the original YOLOv5 and the proposed YOLO-SOD architecture, has been added to comply this comment.

Reviewer 1, Comment 7

The current architectural description supports this information but makes the reader reconstruct it themselves. This comment has been addressed in the response of Comment 6 of the reviewer.

Reviewer 1, Comment 8

Explain the fuzzy membership functions quantitatively.

The five fuzzy rules are given, but the manuscript does not adequately specify the membership functions/ranges for "low," "medium," and "high" cluster number/density. Add a small table specifying:

- input variables,

- membership ranges,

- membership-function type,

- output patch sizes,

- defuzzification method. Table 5: Quantitative specification of the fuzzy inference system used for adaptive patch selection, has been added to address the reviewer’s comment.

Reviewer 1, Comment 9

Tone down unsupported statements.

For example: "current state-of-the-art deep models ... may fail to detect the faces in the wild" and "conventional state-of-the-art networks often fail." These are stronger than necessary. Replace with evidence-based wording such as: "Performance may decrease under unconstrained conditions involving small face size, occlusion, pose variation, and complex backgrounds." The manuscript itself identifies these conditions as challenges. The highlighted statements have been toned down as suggested by the reviewer.

Reviewer 1, Comment 10

Replace subjective wording.

Phrases such as:

- "In our opinion"

- "One sentence answer is..."

- "very good clues"

- "golden 80% accuracy mark"

- "intelligent, cluster-aware modification"

- "promising transfer"

should be changed to objective scientific language. For example, "golden 80% accuracy mark" should simply be removed. The subjective wordings has been replaced with scientific language as suggested by the reviewer.

Reviewer 1, Comment 11

Strengthen the limitations section.

The manuscript acknowledges that far-distance faces were excluded because of poor image quality. This should explicitly appear in the Limitations section, together with:

- dependence of the Cr-based map on illumination/skin-color characteristics,

- possible false clusters caused by background regions,

- limited size of the proposed local dataset,

- lack of evaluation for extremely distant faces. Limitations Section has been added as suggested by the reviewer.

Reviewer 1, Comment 12

Clarify the local dataset methodology.

Include, if available:

- where/how images were collected,

- collection period,

- camera/device information,

- selection criteria,

- annotation procedure,

- annotator verification,

- whether the dataset is publicly available.

This is particularly important because the local dataset is presented as a contribution. Statements about the data collection has been added in Section E of Proposed Methodology titled as ‘Proposed dataset highlighting local attire conditions’ as suggested by the reviewer.

Reviewer 1, Comment 13

Improve the evaluation description.

The manuscript explains mAP/AP at considerable length, but some of the discussion is unnecessarily textbook-like. For a research article, this can be shortened and replaced with the exact evaluation protocol used in the experiments. We thank the reviewer for this valuable suggestion. After careful consideration, we believe that implementing the suggested change may reduce the clarity and interpretability of the manuscript. Therefore, we have retained the current presentation and respectfully hope that the reviewer will understand our rationale.

Reviewer 1, Comment 14

Add an ablation study if the experimental data are available.

This would substantially strengthen the paper without changing the methodology. At minimum:

- baseline YOLOv5

- YOLOv5 + modified architecture

- YOLOv5 + fixed patching

- YOLOv5 + fuzzy patch selection

- complete YOLO-SOD

This would demonstrate whether the improvement comes from the architectural modification, the patching, the fuzzy system, or their combination. We thank the reviewer for this valuable suggestion, which would further strengthen the quality of the work. We plan to conduct this ablation study in our future extension of this research, where the proposed method will be evaluated through hardware implementation.

Reviewer 1, Comment 15

Correct grammar throughout.

There are many recurring grammatical problems, for example:

"These feature maps works perfect..." -> "These feature maps work well..."

"the detection head ... get the feature map" -> "the detection head ... receives the feature map"

"We observed that the accuracy..." -> "We observed that detector accuracy..."

"faces at near to medium distance" -> "faces at near-to-medium distances"

"patches plays an important" -> "patches play an important role" Manuscript has been carefully proofread and grammar has been corrected as suggested by the reviewer.

Reviewer 1, Comment 16

Correct the Conclusion formatting.

The Conclusion currently contains an apparent stray word: "Use This work improves..." This should be removed. Also insert spaces between the reported values: "83.4%84.8%70.4 AP" should be: "83.4%, 84.8%, and 70.4% AP". We think there has been some confusion. We checked thoroughly and found that our submitted manuscript as revision 1 does not contains these errors.

Reviewer 1, Highest-priority fixes

If you want this to remain a minor revision, I would prioritize these five:

1. Fix Table 1 and reconcile the dataset numbers.

2. Resolve 84.8% vs 84.4% for WIDER FACE.

3. Standardize YOLOv5/WIDER FACE/VisDrone/mAP terminology.

4. Add the fuzzy membership-function details.

5. Perform a thorough English/grammar and scientific-style edit.

The underlying contribution - modified YOLOv5 architecture + adaptive patching + Cr-based clustering + FIS - is already clearly described in the manuscript. I would not make major changes to the proposed method unless the reviewer specifically requests additional experiments. We check and rectified all the identified issues as suggest by the reviewer.

C. Response to Reviewer 2

Comment Response and location of revision

Reviewer 2, Comment 1

Please use AP/mAP terminology consistently throughout the manuscript. In particular, Table 9 is titled "Test accuracy" although the reported metric is mAP50, and there are still sentences that describe mAP50 values as "accuracy." These should be revised simply to report the results as mAP50. We thank the reviewer for this comment. We have correctly reported the results as mAP@50 instead of writing accuracy as suggested by the reviewer.

Reviewer 2, Comment 2

The interpretation of the ablation results should be slightly moderated. The modified YOLOv5 architecture provides the dominant improvement, while the additional fuzzy Cr-based patching stage gives a smaller incremental gain. Therefore, statements such as "each module improves the accuracy" should be rephrased so that they accurately reflect the values in Tables 7 and 10. I do not consider additional experiments essential here; a more precise discussion of the reported results would be sufficient. We thank the reviewer for this comment. We have moderated the discussions on the mentioned places of the reviewer.

Reviewer 2, Comment 3

A final careful language edit is still recommended. There are several remaining grammatical and typographical issues (for example, expressions such as "fastly trainable," "the background strengthen," and similar wording). Cleaning these up would improve the readability and presentation of the final manuscript. We thank the reviewer for this comment. We have carefully corrected and cleaned up the grammatical and typographical errors as suggested by the reviewer.

Sincerely,

Ms. Aniqa Saba,

Prof. Dr. Muhammad Faizan Shirazi,

Prof. Dr. Yawar Rehman,

Prof. Dr. Muhammad Mubashir Khan

Attachment Submitted filename: PONE-D-25-65594R1_Minor_Revision_Response_Template.docx

# Editor report: Decision Letter 2

**DOI:** 10.1371/journal.pone.0360794.r005

**Authors:** Usman Ghafoor

1 Oct 2026

YOLO-SOD: Color probability map-based fuzzy framework using deep models for face detection

PONE-D-25-65594R2

Dear Dr. Shirazi,

We’re pleased to inform you that your manuscript has been judged scientifically suitable for publication and will be formally accepted for publication once it meets all outstanding technical requirements.

Within one week, you’ll receive an e-mail detailing the required amendments. When these have been addressed, you’ll receive a formal acceptance letter and your manuscript will be scheduled for publication.

An invoice will be generated when your article is formally accepted. Please note, if your institution has a publishing partnership with PLOS and your article meets the relevant criteria, all or part of your publication costs will be covered. Please make sure your user information is up-to-date by logging into Editorial Manager at Editorial Manager® and clicking the ‘Update My Information' link at the top of the page. For questions related to billing, please contact billing support.

If your institution or institutions have a press office, please notify them about your upcoming paper to help maximize its impact. If they’ll be preparing press materials, please inform our press team as soon as possible -- no later than 48 hours after receiving the formal acceptance. Your manuscript will remain under strict press embargo until 2 pm Eastern Time on the date of publication. For more information, please contact onepress@plos.org.

Kind regards,

Usman Ghafoor

Academic Editor

PLOS One

Additional Editor Comments (optional):

The authors have improved the manuscript based on reviewers' comments.

Reviewers' comments:

# Editor report: Acceptance letter

**DOI:** 10.1371/journal.pone.0360794.r006

**Authors:** Usman Ghafoor

PONE-D-25-65594R2

PLOS One

Dear Dr. Shirazi,

I'm pleased to inform you that your manuscript has been deemed suitable for publication in PLOS One. Congratulations! Your manuscript is now being handed over to our production team.

At this stage, our production department will prepare your paper for publication. This includes ensuring the following:

* All references, tables, and figures are properly cited

* All relevant supporting information is included in the manuscript submission,

* There are no issues that prevent the paper from being properly typeset

You will receive further instructions from the production team, including instructions on how to review your proof when it is ready. Please keep in mind that we are working through a large volume of accepted articles, so please give us a few days to review your paper and let you know the next and final steps.

Lastly, if your institution or institutions have a press office, please let them know about your upcoming paper now to help maximize its impact. If they'll be preparing press materials, please inform our press team within the next 48 hours. Your manuscript will remain under strict press embargo until 2 pm Eastern Time on the date of publication. For more information, please contact onepress@plos.org.

You will receive an invoice from PLOS for your publication fee after your manuscript has reached the completed accept phase. If you receive an email requesting payment before acceptance or for any other service, this may be a phishing scheme. Learn how to identify phishing emails and protect your accounts at https://explore.plos.org/phishing.

If we can help with anything else, please email us at customercare@plos.org.

Thank you for submitting your work to PLOS One and supporting open access.

Kind regards,

PLOS One Editorial Office Staff

on behalf of

Dr. Usman Ghafoor

Academic Editor

PLOS One
